# Fecal Microbiota and Performance of Dairy Cattle from a West Mexican Family Dairy Farm Supplemented with a Fiber-Degrading Enzymatic Complex

**DOI:** 10.3390/vetsci12060518

**Published:** 2025-05-25

**Authors:** José Martín Ruvalcaba-Gómez, Ramón Ignacio Arteaga-Garibay, Luis Miguel Anaya-Esparza, Lorena Jacqueline Gómez-Godínez, Jazmín Guadalupe Martínez-Sotelo, Elías Hernández-Cruz, Luis Eduardo Arias-Chávez

**Affiliations:** 1 Centro Nacional de Recursos Genéticos, Instituto Nacional de Investigaciones Forestales, Agrícolas y Pecuarias, Boulevard de la Biodiversidad 400, Tepatitlán de Morelos 47600, Jalisco, Mexico; arteaga.ramon@inifap.gob.mx (R.I.A.-G.); gomez.lorena@inifap.gob.mx (L.J.G.-G.);; 2 Centro de Estudios para la Agricultura, la Alimentación y la Crisis Climática, Centro Universitario de los Altos, Universidad de Guadalajara, Tepatitlán de Morelos 47620, Jalisco, Mexico; luis.aesparza@academicos.udg.mx; 3 Campo Experimental Centro Altos de Jalisco, Instituto Nacional de Investigaciones Forestales, Agrícolas y Pecuarias, Boulevard de la Biodiversidad 2470, Tepatitlán de Morelos 47714, Jalisco, Mexico

**Keywords:** non-starch polysaccharidases, amplicon-based sequencing, fiber

## Abstract

Non-starch polysaccharide-degrading enzymes are widely used as feed additives in monogastric and ruminant species, with positive effects reported. In this study, the commercial, fiber-degrading enzyme complex Hostazym^®^ X, derived from *Trichoderma citrinoviride* (DSM34663), was included in the total mixed rations of 17 mid-lactating (135 ± 61 days in milk) Holstein cows for 10 weeks. A control group (*n* = 17) was included. Dry matter intake (DMI), milk yield, 4% fat-corrected milk, solid yield, and milk fatty acid profile were assessed. The structure and composition of fecal bacterial communities, as well as PICRUSt2-based functional prediction of bacterial communities, were also evaluated. Higher DMI and milk yield scores were observed in the supplemented group (27.20 vs. 26.59 kgDM/cow/d; and 39.01 vs. 36.70 L/cow/d, respectively). No effects were observed in fat yield, contrary to lactose and protein, which were greater in the supplemented group compared to the control group (1.18 vs. 1.13 and 1.83 vs. 1.75 kg/cow/d, respectively; *p* < 0.05). Palmitic and oleic acids, in addition to monounsaturated fat in milk, were increased in the supplemented group (*p* > 0.05). Enzyme supplementation increased the Patescibacteria (*p* < 0.5) and Actinobacteriota (*p* > 0.05) in feces, but slightly reduced the Bacteroidota and Firmicutes. The *Turicibacter* genus remained at a lower relative abundance after supplementation but *Candidatus_Saccharimonas, Clostridioides, Prevotellaceae* UCG 003, *Corynebacterium, Akkermansia, Syntrophococcus, Erysipelotrichaceae* UCG 008, other *Lachnospiraceae*, other members of the *Eubacterium_coprostanoligenes_group, Bifidobacterium, Rumminococcus, Akkermansia*, and other *Spirochaetaceae* increased, modifying the functional predicted profile of bacterial communities. In conclusion, a positive effect on performance and milk composition were observed through modulation of microbiota induced by enzyme supplementation. The enzyme complex could be a viable supplement alternative in the feeding of dairy cows in semi-intensive productive systems, mainly when an ad libitum feeding scheme is used.

## Simple Summary

A commercial fiber-degrading enzyme complex derived from *Trichoderma citrinoviride* (DSM34663) was included in the total mixed rations of 17 mid-lactating Holstein cows for 10 weeks. Higher DMI and milk yield scores were observed in the supplemented group, and some effects were recorded in lactose, protein, palmitic acid, oleic acid, and monounsaturated fat in milk. However, no effects were observed in fat yield The enzyme supplementation also induced fecal microbiota modulation, modifying the predicted functional profile of bacterial communities.

## 1. Introduction

Cereal co-products have a low starch content and a high crude fiber concentration, including non-starch polysaccharides (NSPs) such as cellulose, hemicellulose, and pectins as the most abundant, as well as fructans, glucomannans, and galactomannans in smaller abundances [1]. These NSPs can be classified as soluble, increasing the viscosity of cud through water absorption, and insoluble, not affecting digesta viscosity, but serving as bulking agents [2]. Nevertheless, digesta viscosity changes negatively affect how endogenous enzymes interact with their targets, with some physiological implications, such as reducing fat solubilization and protein hydrolysis, among others [3]. However, NSPs could be a good energy source due to the degradation of polysaccharides into mono-, di-, or oligosaccharides by ruminal microbiota [4]. Indeed, a considerable amount of NSPs are fermented to produce short-chain fatty acids when they reach the colon, owing to the effect of resident microorganisms [5], which have various health benefits, such as regulating pH and inhibiting the proliferation of potentially pathogenic microorganisms [5,6]. To enhance NSP digestion, non-starch polysaccharide-degrading enzymes have been widely used as feed additives in monogastric and ruminant species. The in vitro efficiency of these fibrolytic enzymes has been assessed, demonstrating increases in feed-fiber degradation [7], which could explain the observed effects in supplemented cattle, including an increase in feed intake and total track digestibility, mainly at low inclusion levels [8]. Fibrolytic enzymes have also exhibited benefits in terms of milk yield, such as those reported by Kung et al. in 2002 [9], who reported that cellulolase and xylanase supplementation in cows increased milk production by up to 3.5%.

On the other hand, reports are inconsistent regarding the benefits associated with non-starch polysaccharides degrading enzyme supplementation. Yang et al., in 2011 [10], reported no effects on the milk production of lactating dairy cows. However, they reported reduced methane-estimated production and increased volatile fatty acid concentration, among other effects, such as an increase in the total digestibility of dry matter and crude protein. Additionally, they reported that production benefits increased when more concentrates were fed. Similarly, Yang et al., in 2019 [11], reported no effects of xylanase supplementation on dry matter intake, milk yield, milk composition (fat, protein, and lactose), and nutrient digestibility in dairy cows. Nonetheless, the effects of exogenous fibrolytic enzymes on the ruminal or fecal microbiota remain unclear. Liu et al. [12] observed slight effects on rumen microbiota after 21 days of supplementing early-lactating dairy cows with fibrolytic and amylolytic enzymes, although the richness and diversity of rumen bacteria did not change. For that reason, in this study, we intend to prove the efficiency of an enzyme complex when used in an ad libitum dairy cow feeding scheme in a semi-intensive dairy farm, while maintaining prolonged interaction times between the enzyme complex and the substrates before intake.

This study aimed to assess the effects of Hostazym^®^ X, a commercial, fiber-degrading enzyme complex, on the productivity, milk composition, and fecal microbiota of lactating dairy cows from a west Mexican family dairy farm.

## 2. Materials and Methods

### 2.1. Animals and Groups

The study was conducted on a commercial dairy farm located in Arandas, Jalisco, Mexico. Thirty-four Holstein mid-lactation cows (135 ± 61 days in milk, DIM), daily production of 39.4 ± 7.5 L of milk per cow, and 631 ± 72 kg of body weight, were used. The cows were fed with a totally mixed ration (TMR) for an experimental period of 60 days. The cows were randomly assigned in a completely randomized design to one of two treatment (groups): TC = TMR without the enzymatic complex (control) and TE = TMR with enzymatic complex supplementation (25 g/cow/day). Each group (17 cows per treatment) was housed in a collective pen. The cows were milked twice daily (05:00 and 16:00 h).

### 2.2. Cattle Feeding and Supplementation

The feeding management of commercial dairy cows was not modified and was maintained according to owner management. The diet was formulated to meet the nutritional requirements of the experimental cows, with 51.4% forage and 48.6% concentrate (dry weight basis). Feed ingredients and refusals were sampled weekly and dried to a constant weight using a forced-air oven. Dried samples were milled to pass through a 1 mm screen and used to determine dry matter, organic matter [13], crude protein [14], ether extract, neutral detergent fiber, and acid detergent fiber [15] (Table 1). The commercial fiber-degrading enzyme complex Hostazym^®^ X Microgranulated 15,000 EPU/g derived from *Trichoderma citrinoviride* (DSM34663) (Primary enzymatic activity: endo 1,4 -β Xilanase, EC 3.2.1.8; secondary activity: endo 1,4-β-glucanase (celullase), endo 1,3(4) β-glucanase, α-amilase, protease. Lot number 23080859084, Huvepharma, Sofia, Bulgaria) was used for TE group supplementation. All the cows in the study had a 15-day adaptation period to the diet, followed by a 60-day experimental period. The RTM was prepared daily using a Super Stars Model MV 620 vertical feed mixer (Cuauhtémoc Chihuahua, Mexico). A daily amount of Hostazym^®^ (25 g/cow/day) was mixed with 3 kg of concentrate and then applied to the feed mixer. TMR was offered once daily (10:00 h), considering a 5% feed rejection rate. The amount of feed offered and rejected per pen was recorded daily, and the data were used to estimate the DMI per cow per day, as suggested by Thomas et al. [16] and Colombo et al. [17].

### 2.3. Sample Collection

#### 2.3.1. Milk

Milk production was calculated using individual milk meters (Metatron 12™, Westfalia™, Oelde, Germany) that were placed directly on the milking line. Milk production measurements (kg of milk/cow/day) were recorded weekly from week two, after the start of the trial until week 10. Milk production was recorded two days in a row each week during the morning and evening milking, as suggested by Eslamizad et al. [18] and Sheehy et al. [19]. During milking, milk from each group was received in individual containers per group, from which two samples per group were collected using a previously sanitized stainless steel manual collector. Milk samples were placed in sterile polypropylene jars with a hermetic lid and transported to the laboratory and preserved under refrigeration (7–10 °C) until processing within the next 24 h.

#### 2.3.2. Feces

Fecal samples were collected from the rectum of each animal according to the suggestions in the “Manual for the collection, preservation, and sending of samples to the laboratory for the diagnosis of common animal diseases” [20]. Samples were immediately placed into sterile 50 mL polypropylene tubes with a hermetic screw cap, and transported under refrigeration (7–10 °C) to the laboratory, where they were stored at −80 °C until processing. Sampling was carried out at weeks two, five and ten of the trial.

### 2.4. Analysis Procedures

#### 2.4.1. Milk Composition

The physicochemical composition of milk (fat concentration, protein, lactose, total solids, non-fat solids and minerals) was determined using an ultrasonic milk analyzer (AKM-98 FARM ECO^®^, NV-LAB, Moscow, Russia), following the methodology reported by Villaseñor et al. in 2017 [21]. Samples were analyzed in triplicate.

#### 2.4.2. Milk Fatty Acid Profile

The milk samples were sent to the Jalisco State Center for Research and Assistance in Technology and Design, A.C. (Guadalajara headquarters) for analysis. The milk fatty acid profile (palmitic acid, stearic acid, oleic acid, linoleic acid, linolenic acid, saturated fat, monounsaturated fat, and polyunsaturated fat) was determined by gas chromatography [22].

#### 2.4.3. Fecal Bacterial Communities Study

Metagenomic DNA was extracted and purified from fecal samples using the commercial Quick-DNA™ fecal/soil Microbe Miniprep system (Zymo Research, Irvine, CA, USA) according to the manufacturer’s instructions. Purified DNA was used to construct 16S DNA libraries by PCR amplification of the V2, V3, V4, and V6–V9 hypervariable regions of the 16S rDNA gene using the 16S Metagenomics™ system according to the manufacturer’s instructions (Thermo Fisher Scientific, Waltham, MA, USA) in a Verity™ thermal cycler (Thermo Fisher Scientific, Waltham, MA, USA). An equimolar mixture was prepared from the amplification products, of which 50 ngwas used to construct 16S rDNA libraries using the Ion Plus Fragment Library commercial system and Ion Xpress barcode adapters (Thermo Fisher Scientific). Libraries were purified (Agentcourt AMPure XP system; Beckman Coulter, Brea, CA, USA) and quantified (Bioanalyzer 2100; Agilent Technologies, Santa Clara, CA, USA) to adjust the concentration to 26 pM. Emulsion PCR using a volume of 25 µL of the equimolar mixture for all samples (One-Touch 2, Thermo Fisher Scientific, Waltham, MA, USA) was performed and enriched using the OneTouch Enrichment system (Thermo Fisher Scientific, Waltham, MA, USA). Sequencing was carried out using the Ion S5™ system (Thermo Fisher Scientific, Waltham, MA, USA).

The quality of sequences was evaluated to remove low-quality regions using the Trimmomatic tool [23]. Bioinformatic processing was performed using the nf-core ampliseq pipeline v2.3.2 [24,25]. Sequence visualization and chimeric and low-quality sequences removal were performed using the MultiQC tool and the DADA2 software package, respectively [26,27]. Taxonomic assignment within the DADA2 module was performed using the SILVA v-132 database as a reference (16S rRNA gene sequences clustered at 99% similarity) [28]. Alpha diversity was calculated using the QIIME2 [29] module; meanwhile, distances based on ASV abundance between samples (beta diversity) were measured using the method of “weighted UniFrac” and visualized using principal coordinate analysis (PCoA) plot. Functional prediction based on the 16S DNA sequences was evaluated using the PICRUSt2 (v. 2.4.1) software to explore the possible metabolic mechanisms associated with bacterial communities in fecal samples [30].

### 2.5. Statistical Analysis

Feed intake, production, and milk composition data were analyzed using the MIXED procedure with the REML estimation method, diagonal covariance matrix, and means comparison using Tukey’s (α = 0.05) using the SAS statistical software ver. 9.4 (SAS Institute Inc., Cary, NC, USA). Statistical analysis and results visualization of sequencing data were performed using the Marker Data Profiling module of Microbiome Analyst ver. 2.0 [31]. Alpha-diversity was assessed using the Observed and Chao1 indices, to evaluate the richness of bacterial communities, and the Shannon and Simpson indices, to evaluate the species diversity of the communities. Differences in taxon abundance were corroborated using the Kruskal–Wallis test performed in the SPSS statistical package ver. 29.0.2.0 (IBM Corp. Armonk, NY, USA), using the relative abundance at the Phylum, Family and Genus levels of relative abundance. The relative abundance at the genus level was used to perform principal component analysis in the SPSS statistical software to assess the beta-diversity of the bacterial communities. Pearson’s correlations were calculated to assess the association between the relative abundance of the observed bacterial genera with DMI, milk production, and milk solid yield. Plots were generated using Origin (Pro) software ver. 2024b (OriginLab Corporation, Northampton, MA, USA).

## 3. Results

### 3.1. Dry Matter Intake

Dry matter intake (DMI) was higher (*p* < 0.05) in animals in the TE group that in those in the TC group (Table 2). The effects associated with treatment, time and the interaction of both factors were observed (*p* > 0.05; Table S1, Supplementary Material).

### 3.2. Milk Yield

Significant statistical differences (*p* < 0.05) were observed for average milk production between the treatments (Table 2 and Table S2); however, no effect was observed for the time factor (weeks, *p* > 0.05). Regarding 4% fat-corrected milk (4%FCM), significant effects (*p* < 0.05) were observed between both groups, both associated with the treatment and the evaluation week, as well as by the interaction of both factors.

### 3.3. Milk Composition Profile

Effects associated with the treatment (supplementation with the enzymatic complex) were observed for the parameters of density, lactose, non-fat solids and protein in milk (*p* < 0.05. Table 2 and Table S3), as well as those associated with the time factor (weeks of evaluation) and the interaction of both factors (treatment × week).

Regarding the fatty acid profile in milk (Table 3 and Table S4), significant differences associated with the treatment were observed for the concentration of palmitic acid (*p* < 0.05), which was higher in milk produced by the TE group. Regarding the time factor (evaluation week), the concentration of linolenic acid was higher (*p* < 0.05) in the milk produced by cows in the TE group. Significant differences (*p* < 0.05) were observed in the treatment × week interaction for the concentration of oleic acid and monounsaturated fat, which exhibited higher concentrations in milk from cows supplemented with the enzyme complex.

### 3.4. Fecal Microbiota

**Figure 1:**
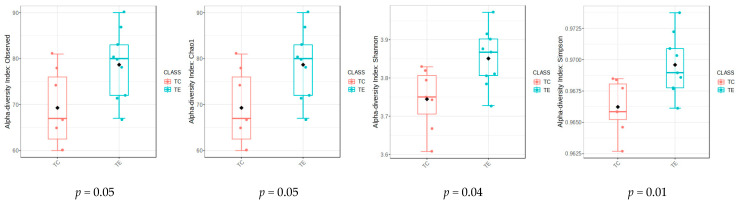
Alpha diversity indices for fecal bacterial communities from lactating cows with (TE) and without (TC) the inclusion of the enzymatic complex.

A total of 2,218,848 readings, obtained from massive amplicon sequencing, were processed after filtering and chimera removal. According to the Kruskal–Wallis pairwise comparison test, significant differences were recorded for the Observed, Chao1, Shannon and Simpson diversity indices (*p* ≤ 0.05, Figure 1).

**Figure 2:**
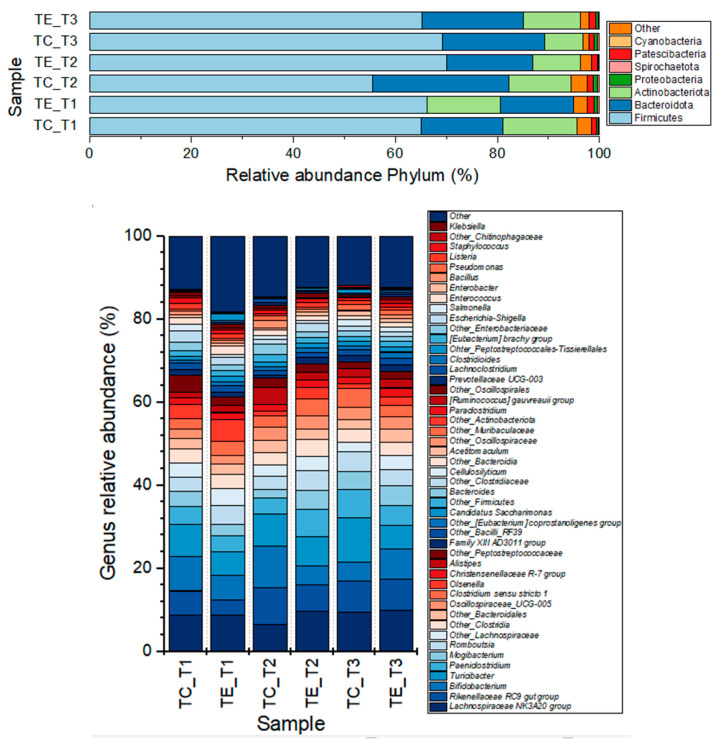
Relative abundance barplot of Phylum and main bacterial genera observed in fecal bacterial communities from lactating cows with (TE) and without (TC) the inclusion of the enzymatic complex, at weeks 2, 5 and 10 (T1, T2, and T3, respectively) of evaluation.

Sequences were taxonomically assigned to 8 bacterial phyla, 16 classes, 115 families, and 257 bacterial genera (Figure 2).

**Figure 3:**
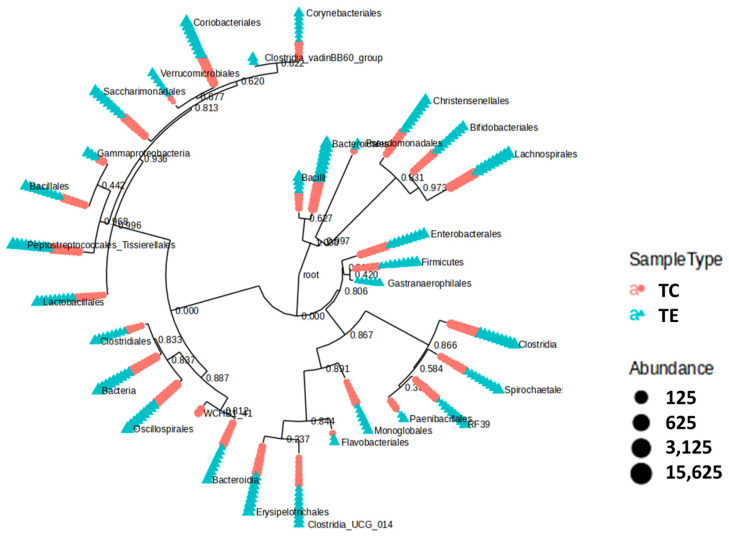
Phylogenetic tree representing the most abundant bacterial orders found in bacterial communities associated with feces from cows supplemented or not supplemented with (TE) or without (TC) the enzymatic complex.

The phylogenetic tree based in the main bacterial observed orders, exhibited differences between fecal bacterial communities from cows within the TE or TC groups (Figure 3).

**Figure 4:**
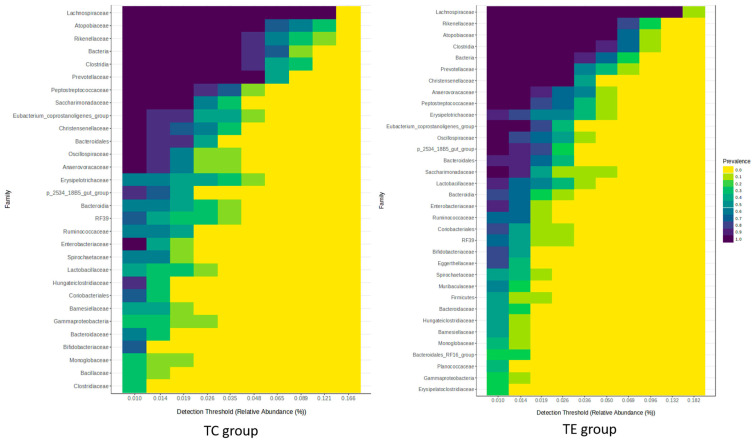
Family core microbiome of fecal bacterial communities from cows supplemented (TE) or non-supplemented (TC) with the enzymatic complex.

The most abundant phylum in all samples was Firmicutes, followed by Bacteroidota and Actinobacteriota (Table S5). Significant differences (*p* < 0.05) were observed only for the Patescibacteria phylum. However, a tendency to decrease its relative abundance was observed for the Bacteroidota phylum in feces from the TE group, while the Actinobacteriota phylum tended to increase (19.96 vs. 20.17, and 11.19 vs. 7.44 of relative abundance at week 10, respectively). The most representative bacterial families in all samples were the *Lachnospiraceae, Peptostreptococcaceae, Erysipelotrichaceae, Bifidobacteriaceae, Rikenellaceae, Anaerovoracaceae, Clostridiaceae, Atopobiaceae*, and *Oscillospiraceae* (Table S6), and significant differences (*p *< 0.05) were observed for the *Saccharimonadaceae*, *Eubacterium_coprostanoligenes* group, *Akkermansiaceae, Corynebacteriaceae*, and *Erysipelotrichaceae* families. The *Bifidobacteriaceae, Atopobiaceae*, and *Monoglobaceae* families were more abundant in feces from the TE group that in the TC group at week 10 (7.29 vs. 4.51, 2.39 vs. 1.45, and 0.20 vs. 0.04%, respectively), as well as other *Enterobacterales* (0.18 vs. 0.00%), although their relative abundance was not significantly different (*p* > 0.05). On the other hand, the *Erysipelotrichaceae, Moraxellaceae*, and *Enterococcaceae* remained in lower relative abundance in feces from supplemented cows in comparison with feces from the control group (6.44 vs. 11.31, 0.04 vs. 0.43, 0.02 vs. 0.24%, respectively). The core microbiome plot corroborates differences in the core microbiome at the family level (Figure 4).

At the genus level, the most representative items were the Lachnospiraceae_NK3A20 group, Bifidobacterium, Turicibacter, Rikenellaceae_RC9 gut group, Paeniclostridium, Mogibacterium, Olsenella, Romboutsia, Catenisphaera, and Clostridium_sensu_stricto_1 (Table S7). However, significant differences among groups (*p* < 0.05) were observed for the Turicibacter, other Lachnospiraceae, Candidatus_Saccharimonas, other Eubacterium_coprostanoligenes_group, Clostridioides, Prevotellaceae_UCG_003, Syntrophococcus, Erysipelotrichaceae_UCG_008, Corynebacterium, Akkermansia, Other Spirochaetaceae, and Other Succinivibrionaceae. After 10 weeks of evaluation, the Turicibacter genus remained at a lower relative abundance in feces from supplemented cows than in the control group (*p* < 0.05; 5.64 vs. 10.78%). Meanwhile, Candidatus_Saccharimonas, Clostridioides, Prevotellaceae_UCG_003, Corynebacterium, Akkermansia, Syntrophococcus, Erysipelotrichaceae_UCG_008, other Lachnospiraceae, other members of the Eubacterium_coprostanoligenes_group, and other Spirochaetaceae increased (*p* < 0.05; 1.33 vs. 1.08, 0.35 vs. 0.15, 0.67 vs. 0.40, 0.07 vs. 0.02, 0.02 vs. 0.00, 0.21 vs. 0.01, 0.04 vs. 0.00, 3.42 vs. 2.34, 1.50 vs. 0.77, and 0.01 vs. 0.00%, respectively). Other genera tended to increased their relative abundance in the TE group when compared with the TC group after 10 weeks of supplementation, although not statistical differences were reached (*p* > 0.05), such as Bifidobacterium (7.28 vs. 4.51%), other Oscillospiraceae (0.95 vs. 0.13%), the Ruminococcus_gauvreauii group (0.82 vs. 0.29%), other Oscillospirales (0.79 vs. 0.33%), other Ruminococcaceae (0.51 vs. 0.23%), and the Eubacterium_brachy group (0.15 vs. 0.04%). On the other hand, other genera not-significantly decreased their relative abundance in feces from TE in comparison with TC (*p* > 0.05), such as Paeniclostridium (4.88 vs. 6.72%), Clostridium_sensu_stricto_1 (2.47 vs. 4.58%), Other Peptostreptococcales_Tissierellales (0.33 vs. 0.81%), Enterococcus (0.02 vs. 0.26%), and Coprobacter (0.006 vs. 0.009%).

**Figure 5:**
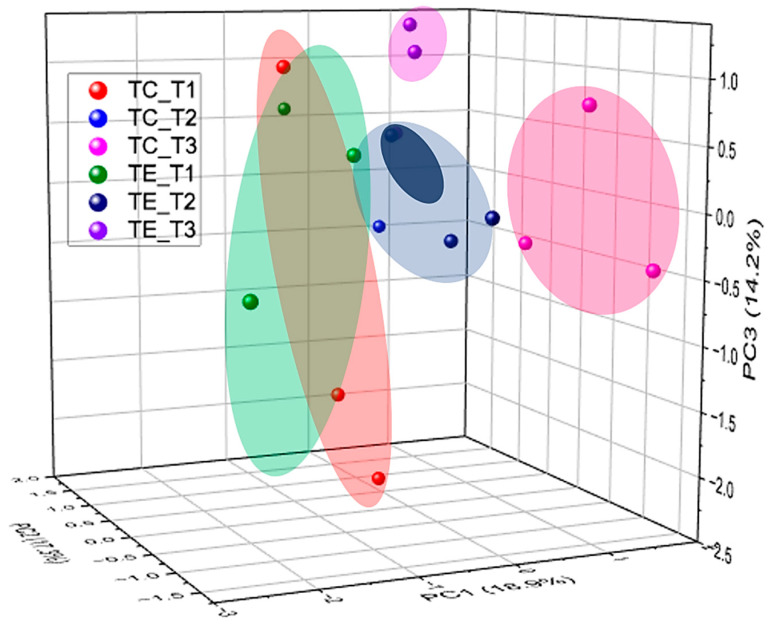
Principal component analysis plot from diversity of bacterial genera observed in fecal microbiota of cows supplemented with the enzymatic complex (TE) and without supplementation (TC) at different sampling times (T1 = week 1, T2 = week 5, T3 = week 10).

Differences in the composition and structure of fecal bacterial communities in TE and TC cows were corroborated by beta-diversity analysis through principal component analysis (PCA, Figure 5).

Significant correlations (*p* < 0.05) were observed among some bacterial genera and DMI, milk production and composition. DMI was positively correlated (*p* < 0.05) with the Christensenellaceae R7 group (R = 0.832), *Caproiciproducens* (R = 0.355), unassigned Succinivibrionaceae (R = 0.904), and Ruminococcaceae CAG 352. Milk yield was positively correlated with unassigned Lachnospiraceae (R = 0.853), unassigned Oscillospirales (R = 0.938), and *Syntrophococcus* (R = 0.823), but negatively correlated (*p* < 0.05) with unassigned Muribaculaceae (R = −0.863), Acinetobacter (R = −0.886), *Enterococcus* (R = −0.932), the *Eubacterium tenue* group (R = −0.923), the *Eubacterium oxidoreducens* group (R = −0.923), and unassigned Saccharimonadales (R = −0.923). Fat yield exhibited positive correlation values (*p* < 0.05) with unassigned Clostridia (R = 0.836), *Syntrophococcus* (R = 0.843), unassigned Coriobacteriales (R = 0.813), unassigned Spirochaetaceae (R = 0.890), the Lachnospiraceae NK4A136 group (R = 0.871), unassigned Microbacteriaceae (R = 0.924), *Lachnospira* (R = 0.871), and *Leucobacter* (R = 0.871). Regarding milk solids, Clostridia positively correlated to protein yield (R = 0.87), *Candidatus_Saccharimonas*, the *Ruminococcus gauvreauii* group, the *Eubacterium hallii* group, unassigned Enterobacterales, the *Eubacterium nodatum_group, Erysipelotrichaceae* UCG 002, and the Prevotellaceae NK3B31 group, negatively correlated with NFS yield (R = −969, −0.844, −0.820, −0.938, −0.884, −0.854, and −0.847, respectively). Lactose yield positively and negatively correlated with some taxa, including *Shuttleworthia, Faecalitalea*, the Lachnospiraceae NK4A136 group, unassigned Microbacteriaceae, *Leucobacter*, and *Lachnospira* (R = 0.820, −0.867, 0.940, 0.946, 0.940, and 0.940, respectively).

**Figure 6:**
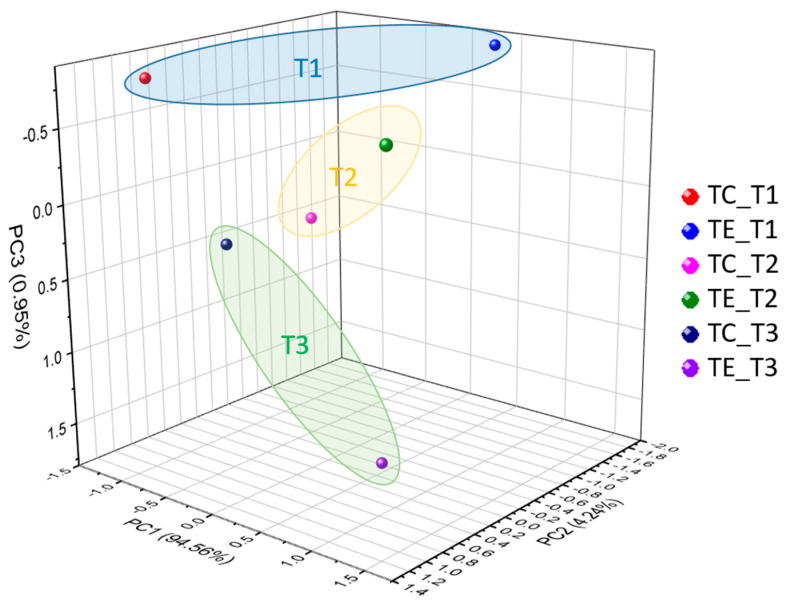
Principal component analysis plot from MetaCyc-predicted pathways from fecal bacterial communities of cows supplemented with the enzymatic complex Hostazym^®^ X (TE) and without supplementation (TC) at different sampling times (T1 = week 1, T2 = week 5, T3 = week 10).

To evaluate the possible changes in the functional profile of fecal bacterial communities due to changes in composition and structure, the functional profile was predicted using PICRUSt2 software [30]. A total of 383 MetaCyc pathways were annotated, of which 49 exhibited differences (*p* < 0.05) in feces between the TE and TC groups regarding the predicted functional potential. The MetaCyc-predicted pathways were observed as different in terms of frequency among the supplemented and the non-supplemented group, included pathways related with the amide, amidine, amine, and polyamine biosynthesis, amino acids biosynthesis, aromatic compound degradation, carbohydrate biosynthesis, carbohydrate degradation, cell structure biosynthesis, cofactor, carrier, and vitamin biosynthesis, cell wall biosynthesis, nucleoside and nucleotide biosynthesis, and secondary metabolite biosynthesis. Principal Component Analysis corroborated differences in the predicted functional profiles of bacterial communities based on the frequency of the predicted pathways (Figure 6).

At week 10 of the evaluation, 16 pathways increased their frequency of prediction in feces from the TE group compared with the TC group (*p* > 0.05). These predicted pathways were associated with secondary metabolites biosynthesis (PWY-7255); aromatic compounds degradation (PWY-5180, PWY-5182); generation of precursor metabolites and energy (PWY-7007); cofactors, prosthetic groups, and electron carriers biosynthesis (PWY-6383, PWY1G-0); amino acids biosynthesis (PWY-7527); cell structures biosynthesis (PWY-6397); and nucleosides and nucleotides biosynthesis (PWY-7210, PWY-7198, PWY-4361). However, at least 70 other pathways were more frequently predicted in feces from the TE group at week 10, mostly representing amino acids biosynthesis, carbohydrates biosynthesis, carbohydrates degradation, cell structures biosynthesis, fatty acid and lipid biosynthesis, cofactors, prosthetic groups, electron carriers biosynthesis, generation of precursor metabolites and energy, and nucleosides and nucleotides biosynthesis.

## 4. Discussion

Exogenous enzymes contribute to non-starch polysaccharide degradation, and thus, the liberation of oligosaccharides with prebiotic potential can enhance the productivity performance of cows [1,5]. In this study, we supplemented mid-lactation dairy cows allocated in a commercial production unit from a west Mexican family dairy farming. These results suggest that the commercial enzymatic complex, derived from the *Trichoderma citrinoviride* DSM34663 strain, improves the DMI and productivity of cows while increasing the daily yield of fat, protein, lactose and NFS.

The evidence regarding the effects of exogenous enzymes is diverse. Some reports indicate that fibrolytic enzymes do not affect milk yield and composition [32,33,34]. For example, in 2019, Yang et al. supplemented lactating dairy high-producing dairy cows with a coated thermotolerant enzyme formulation (endo-1,4-β-xylanase derived from *Aspergillus oryzae* with no cellulosic or proteolytic activities), added during the pelletization of the concentrate (1.5 g of a 1000 units of fungal xylanase per g/kg of total DM intake). The authors reported no changes (*p* > 0.05) in DMI, milk yield, and milk composition (fat, protein and lactose, percentage in milk and daily yield). The study neither report changes in the milk fatty acid profile associated with xylanase supplementation [11].

On the other hand, Liu et al. in 2022 [12], reported an increase of 3.5% FCM and energy-corrected milk (*p* < 0.05) in early lactation Holstein cows (90 ± 5 DIM) after 28 d of supplementation with a mixture of fibrolytic and amylolytic enzymes (70 g/cow/d; 3500 CU/g of cellulase, 2000 XU/g of xylanase, 17,500 GU/g of β-glucanase, and 37,000 AU/g of amylase). The authors highlighted the increase in α-amylase and xylanase activities, as well as the ammonia-N concentration (*p* < 0.05), in addition to a tendency to increase β-glucanase activity (*p* = 0.08) in the ruminal fluid. Additionally, Savela et al. in 2022 [35] reported an increase in milk yield (*p* = 0.05), but milk fat reduction (*p* = 0.01), through supplementation with a fibrolytic enzyme (xylanase, endoglucanase, and exoglucanase activities; 10 g enzyme/cow/day) in early lactation Holstein cows (40 ± 14 DIM, even when no effect on DIM (*p* > 0.05) was observed. Similarly, in another study, a commercial enzyme mixture produced by *Trichoderma reesei* (8000 units of endo-1,4-ßglucanase, 18,000 units of endo-1,3(4)-ß-glucanase, and 26,000 units of 1,4-ßxylanase per mL) was supplemented for 56 days to early (50 ± 16 DIM) and mid-lactation (136 ± 26 DIM) Holstein cows (3.8 and 3.9 mL/kg-TMR-DM, respectively). The authors reported no differences (*p* > 0.05) in DMI, milk yield, 4% FCM, and energy-corrected milk, or concentration of milk fat and protein; nonetheless, the authors pointed out that, in mid-lactation cows, a tendency to increase ECM yield was observed (*p* = 0.09) [36]. However, even when effects on milk yield are not consistent when fibrolytic enzymes are supplemented, it has been reported that their use can enhance the synthesis of short-chain fatty acids in the rumen, with no effects on ruminal pH but effects on ruminal NH3-N, DMI, chewing time, and ruminating [32].

Milk fat is a combination of monounsaturated, polyunsaturated, and saturated fatty acids, and it have been described around 400–500 fatty acids in milk fat [37] as a result of the microbial-mediated dehydrogenation of polyunsaturated fatty acids consumed by cows. Strategies for improving the milk fatty acid profile, mainly by increasing the concentration of poly- and monounsaturated fatty acids, are commonly based on the inclusion of green forages in cow feed rations [38,39]. Our results demonstrated that supplementation of cows with the enzymatic complex resulted in a higher concentration of oleic acid and monounsaturated fat, but a lower concentration of linolenic acid in milk compared to the control group (*p* < 0.05). Similar results were reported for goats supplemented with cellulose (2 mL/kg/DMI) for 70 days, resulting in lower linoleic and linolenic acid content and saturated fatty acids in comparison with milk from non-supplemented goats [40].

Several factors can define the fecal cattle microbiota, including fecal starch concentration and feeding operations [41]. Our study suggests fecal microbiota modulation due to enzyme complex supplementation, which increases or reduces the relative abundance of certain bacterial groups (*p* < 0.05), possibly due to the release of prebiotic compounds from fiber degradation. In contrast, in a previously reported study, there were no significant differences in terms of bacterial richness and diversity between early lactation dairy cows (90 ± 5 DIM) supplemented and non-supplemented with a mixture of fibrolytic and amylolytic enzymes [12]. The authors only observed a tendency of differentiation through the PCA (R = 0.22, *p* = 0.098), resulting in differences, in terms of abundance, for ten bacterial genera (compared to the control group, abundance reduction of: *unclassified_f_Veillonellaceae, norank_f_Veillonellaceae, Syntrophococcus, Selenomonas* and *Lachnospiraceae_UCG-001*; and an increase in abundance of *Ruminococcaceae_UCG-001, Hydrogenoanaerobacterium, Sphaerochaeta, Veillonellaceae_UCG-001, and [Eubacterium]_nodatum_group*.). On the other hand, supplementation with a mixture of cellulase, xylanase, β-glucanase, and mannanase in cow feed rations [42] increased the Simpson diversity index values, while the Shannon index decreased when 0.1% of the enzymatic mixture per kg of DM was added to the TMR of 5-month old female fattening hybrid sheep. These changes in diversity were associated with a lower relative abundance of ruminal Proteobacteria but a higher abundance of the *Prevotella* and *Rumminococcus* genera, in addition to increasing NH3-N concentration in the rumen, which indirectly promotes the dietary protein and NSP degradation, as proposed by the authors.

In this study, significant differences (*p* < 0.05) were observed for Patescibacteria, a phylum associated with anoxic environments, such as underground water [43], which exhibited higher relative abundances in feces from cows in the TE group. No information has been reported on the function of the phylum Patescibacteria; however, it has been previously reported as part of the rumen and fecal microbiota, as well as milk microbiota from cows with low somatic cell count levels [44]. At the genus level, the relative abundance of *Eubacterium_coprostanoligenes* increased in the feces of the TE group. This genus has been observed to increase in cattle with a high milk yield, which may be associated with increases in ruminal pH and enhanced fermentation patterns. Although its function is still unclear [45], it has been linked to the regulation of lipid metabolism [46]. *Candidatus Saccharimonas*, which also seems to increase its relative abundance associated with the enzyme supplementation (*p* < 0.05), and has been reported in young calves with a lower abundance of rumen methanogen populations when an anti-methanogen compound was used, induces changes in fermentation from acetate to propionate and the increasing of branched-chain fatty acids [47].

*Prevotellaceae* UCG_003 also showed a higher relative abundance in feces from cows belonging to the TE group (*p* < 0.05), and it has been associated with organic acid production [48]. In addition, it has been reported that *Prevotella* possesses multiple esterase activities that allow for carbohydrate metabolism, including Axe1-6A, AxeA1, and Axe7A esterase, which are related to the metabolism of xylo-oligosaccharides, and that synergistically work with xylan-depolymerizing enzymes (e.g., xylanases) [49]. Additionally, it has been reported that *Prevotella* exhibits a versatile behavior in the use of nitrogen, contributing to protein degradation [50].

*Turicibacter*, which exhibits a significantly lower relative abundance in feces from the TE group, is a bacterial genus reported to be one of the predominant genera in feces [51] (Liu et al., 2015). This genus has been genetically related to *Paeniclostridium, Romboutsia*, and *Clostridium sensu stricto 1*, which were also observed in lower proportions after 10 weeks of supplementation in comparison to feces from the control group, and are related to anaerobic digestion of bovine manure and some volatile fatty acids [52,53]. Studies on the functionality of *Turicibacter* remain scarce; however, it has been pointed out that it participates in bile acid and lipid metabolism [54]. Similarly, *Paeniclostridium* and *Clostridium* have been reported to contribute to the formation of secondary bile acids, as well as *Romboutsia*, which might also be involved in glucose and lipid metabolism [55]. *Syntrophococcus*, a genus reported to be predominant in the rumen of healthy cows [56], also showed higher abundances in feces from the supplemented cows. The *Syntrophococcus* genus is an acid-acetic-producing bacterium that may contribute to the settlement and enhancement of rumen function [56,57]. In this context, a lower pH could occur in supplemented cows, which could also be related to the increase in *Erysipelotrichaceae* UCG 008, which has been linked to low ruminal pH and fermentable diets, as well as *Candidatus Saccharimonas* [58].

Although it was detected in a very low proportion, the *Corynebacterium* genus was observed in higher relative abundance in feces from the TE group than in the TC group. *Corynebacterium* has been reported in cows with a risk of mastitis [59]. Nonetheless, *Corynebacterium* also has been reported as a member of raw milk microbiota that remain present during cheese preparation and maturation [60] and certain rumen-associated strains, have exhibited beneficial potential, such as bile salts deconjugation and lactose degradation and assimilation [61]. Even when observed in very low proportions, the *Akkermansiaceae* family reported higher relative abundances in feces from the TE group (*p* < 0.05) and its member, the genus *Akkermansia*. *Akkermansia* has been reported as a member of the ruminal and fecal microbiota, with higher relative abundances in feces than in the rumen, and has been associated with glycan biosynthesis and metabolism [62]. Some unassigned *Lachnospiraceae* members remained at a higher proportion in the feces of the TE group. Some genera belonging to *Lachnospiraceae*, the most abundant family observed in all samples, have been linked to cellulose and pectin degradation, including the *Butyrivibrio* and *Lachnospira* genera [63]. Finally, we also found—in very low proportions, but more abundant during enzyme supplementation—unassigned members of the *Spirochaetaceae* family, which are frequently found in cow feces and are related to fiber degradation [64,65].

*Bifidobacterium*, as well as unassigned *Oscillospiraceae*, the *Ruminococcus gauvreauii* group, unassigned *Ruminococcaceae*, and the *Eubacterium brachy* group, were observed to increase at week 10 after enzyme supplementation, even when there were no significant differences (*p* > 0.05). *Bifidobacterium* is considered a beneficial genus, is widely used as a probiotic, and is proposed to be a main representative of fecal cow microbiota, increasing in high-productive animals [66]. The *Oscillospiraceae* family has been related to growth performance and immunity in sheep [67]; it is considered a butyrate-producer [68] and potentially offers benefits such as the production of bile acids and provides protection against pathogens [69,70]. On the other hand, *Ruminococcaceae* is a family commonly found in cow feces, and some of its genera are distinguished as fibrolytic and contribute to the formation of short-chain fatty acids [71,72].

The observed changes in the microbiota structure associated with feces from the enzyme-supplemented cows matched their predicted functional profile, which exhibited changes in metabolic predicted pathway frequency, mainly involved in the metabolism of carbohydrates and proteins, secondary metabolite biosynthesis, and the generation of precursor metabolites and energy. Changes in the predicted functional profile could explain the changes in the productive performance of cows and milk composition, which were enhanced by enzyme supplementation.

Finally, it is important to consider that the effects of fibrolytic enzymes are dependent on several factors that define their level of hydrolytic action, such as the length of the substrate chains that it hydrolyzes— to the shorter the chain length, the lower the hydrolytic activity exhibited by the enzyme [73]. The optimal dose of the enzyme could also fluctuate depending on factors associated with the animal and the availability and characteristics of the substrate to be hydrolyzed. Sensitivity to endogenous inhibitors, grade of selectivity, carbohydrate binding sites [74], the concentration and availability of fiber in feed [75,76], parity, physiological stage, and the microbial fermentation pattern [76], among others, could explain the diversity of results reported for the use of fiber-degrading enzymes in the feeding of dairy cows. Additionally, fiber-degrading enzymes seem more effective when the feeding of the cows is maintained *ad libitum*, in comparison with restricted intake systems, which could be associated with extended ruminal retention periods, increasing fiber digestion. In addition, the pre-ingest hydrolysis of fiber could be enhanced by the extended contact time in the *ad libitum* scheme, as proposed by some authors [75,77,78]. However, under the conditions in which the study was carried out, positive effects on productivity were observed, accompanied by changes in milk quality and fecal microbiota.

## 5. Conclusions

Using a commercial fiber-degrading enzyme complex derived from *Trichoderma citrinoviride* (DSM34663) to supplement mid-lactation Holstein cows improved productivity performance and milk composition. The observed changes could be related to a prebiotic effect induced by enzyme supplementation that modifies the structure of fecal microbiota as an indicator of ruminal microbiota modulation and, consequently, the fermentation pattern, favoring the production of compounds such as secondary metabolites and energy.

## Figures and Tables

**Table 1:** Ingredients and average chemical composition of diets (dry basis).

	Treatment (Group)	
	TC (Control)	TE (ECS)
**Trait (%, DB)**		
Corn silage	31.7	31.7
Alfalfa hay	7.9	7.9
Corn stover	11.9	11.9
Concentrate ^1^	48.6	48.6
Enzyme complex (g/cow)	0	25
**Composition (g/Kg)**		
Dry matter	567.2	565.5
Organic matter	915.0	916.4
Crude protein	158.7	161.7
Ether extract	36.1	35.9
Neutral detergent fiber	350.7	346.5
Acid detergent fiber	245.3	241.2

^1^ Concentrate composition: 31% rolled corn, 21% soybean meal, 16.5% canola meal, 13% ground corn, 10% wheat bran, 4.5% distiller’s dried grain, 1.5% overage fat, and 2.5% vitamin mineral premix. ECS: Enzymatic complex supplementation.

**Table 2:** Dry matter intake, milk yield, and milk composition of cows with and without the inclusion of the enzymatic complex.

	Group (Treatment)			Significance (*p*-Value)		
Item	TC (Control)	TE (ECS ^1^)	SEM	Treatment	Week	Treat × Week
DMI, KgDM/cow/d *	26.59	27.20	0.092	**<0.0001**	**0.0008**	**0.0003**
Milk yield, kg/d	36.70	39.01	0.605	**0.007**	0.306	0.981
4%FCM, kg/d	32.54	34.11	0.533	**0.039**	0.519	0.975
Milk fat, %	3.25	3.16	0.050	0.226	0.981	0.670
Milk fat yield, kg/cow/d	1.19	1.23	0.019	0.112	0.739	0.781
Milk protein, %	3.08	3.03	0.004	**<0.0001**	**<0.0001**	**<0.0001**
Milk protein yield, kg/cow/d	1.13	1.18	0.001	**<0.0001**	**<0.0001**	**<0.0001**
Milk lactose, %	4.78	4.70	0.006	**<0.0001**	**<0.0001**	**<0.0001**
Milk lactose yield, kg/cow/d	1.75	1.83	0.002	**<0.0001**	**<0.0001**	**<0.0001**
Milk NFS, %	8.65	8.50	0.009	**<0.0001**	**<0.0001**	**<0.0001**
Milk NFS yield, kg/cow/d	3.17	3.31	0.004	**<0.0001**	**<0.0001**	**<0.0001**
Milk density, kg/L	1.0288	1.0286	0.399	**<0.0001**	**<0.0001**	**<0.0001**

^1^ ECS: Enzymatic complex supplementation. * DMI measured at the pen level. FCM: 4% fat corrected milk. NFS: Non-fat solids. Bold formatting: significant differences.

**Table 3:** Milk fatty acid profile of cows with and without inclusion of the enzymatic complex.

	Group (Treatment)			Significance (*p*-Value)		
Component, g/L	TC (Control)	TE (ECS)	SEM	Treatment	Week	Treat × Week
Palmitic acid	11.984	12.567	0.171	**0.029**	0.475	0.336
Estearic acid	3.550	3.326	0.197	0.437	0.491	0.481
Oleic acid	8.137	8.491	0.124	0.066	0.145	**0.015**
Linoleic acid	0.921	0.923	0.018	0.950	0.722	0.610
Linolenic acid	0.161	0.159	0.003	0.721	**0.024**	0.451
Saturated fat	14.615	15.891	0.654	0.193	0.383	0.251
Monounsaturated fat	8.137	8.491	0.124	0.066	0.145	**0.015**
Polyunsaturated fat	1.082	1.081	0.021	0.978	0.530	0.567

ECS: Enzymatic complex supplementation.

## Data Availability

The derived 16S rRNA gene sequences are available at the NCBI under the Bioproject ID PRJNA1220145.

## Supplementary Materials

The following supporting information can be downloaded at: https://www.mdpi.com/article/10.3390/vetsci12060518/s1, Table S1: Dry matter intake of cows with and without the inclusion of the enzymatic complex. Table S2: Milk production (total and corrected to 4% fat) of cows with and without the inclusion of the enzymatic complex. Table S3: Physicochemical composition profile of milk produced by cows with and without the inclusion of the enzymatic complex. Table S4: Fatty acid profile in milk produced by cows with and without the inclusion of the enzymatic complex. Table S5: Main observed Phyla in fecal bacterial populations from feces of cows with and without the inclusion of the enzymatic complex. Table S6: Relative abundance of the families of bacteria observed in the fecal microbiota of cows supplemented with the enzymatic complex (TE) and without supplementation (TC) at different sampling times (T1 = week 1, T2 = week 5, T3 = week 10). Table S7: Relative abundance of the families of bacteria observed in the fecal microbiota of cows supplemented with the enzymatic complex (TE) and without supplementation (TC) at different sampling times (T1 = week 1, T2 = week 5, T3 = week 10).

## References

[1] Habte-Tsion H.M., Kumar V., Nunes S., Kumar V. (2018). Nonstarch Polysaccharide Enzymes-General Aspects. Enzymes in Human and Animal Nutrition: Principles and Perspectives.

[2] Davidson M.H., McDonald A. (1998). Fiber forms and functions. Nutr. Res..

[3] Smits C.H.M., Annison G. (1996). Non-Starch Plant Polysaccharides in Broiler Nutrition-towards a Physiologically Valid Approach to Their Determination. J. World Poult. Sci..

[4] Jurkovich V., Brydl E., Rafai P. (2002). Effects of a Non-Starch Polysaccharidase Enzyme Preparation from *Thermomyces lanuginosus* on Energy and Protein Metabolism and Milk Yield of Dairy Cattle. Acta Vet. Hung..

[5] Kumar V., Sinha A.K., Makkar H.P.S., de Boeck G., Becker K. (2012). Dietary Roles of Non-Starch Polysachharides in Human Nutrition: A Review. Crit. Rev. Food Sci. Nutr..

[6] Scott K.P., Duncan S.H., Flint H.J. (2008). Dietary Fibre and the Gut Microbiota. Nutr. Bull..

[7] Bontà V., Battelli M., Rama E., Casanova M., Pasotti L., Galassi G., Colombini S., Calvio C. (2024). An In Vitro Study on the Role of Cellulases and Xylanases of *Bacillus subtilis* in Dairy Cattle Nutrition. Microorganisms.

[8] Beauchemin K.A., Rode L.M., Maekawa M., Morgavi D.P., Kampen R. (2000). Evaluation of a Nonstarch Polysaccharidase Feed Enzyme in Dairy Cow Diets. J. Dairy Sci..

[9] Kung L., Cohen M.A., Rode L.M., Treacher R.J. (2002). The Effect of Fibrolytic Enzymes Sprayed onto Forages and Fed in a Total Mixed Ratio to Lactating Dairy Cows. J. Dairy Sci..

[10] Arriola K.G., Kim S.C., Staples C.R., Adesogan A.T. (2011). Effect of Fibrolytic Enzyme Application to Low- and High-Concentrate Diets on the Performance of Lactating Dairy Cattle. J. Dairy Sci..

[11] Yang Y., Ferreira G., Corl B.A., Campbell B.T. (2019). Production Performance, Nutrient Digestibility, and Milk Fatty Acid Profile of Lactating Dairy Cows Fed Corn Silage- or Sorghum Silage-Based Diets with and without Xylanase Supplementation. J. Dairy Sci..

[12] Liu Z.K., Li Y., Zhao C.C., Liu Z.J., Wang L.M., Li X.Y., Pellikaan W.F., Yao J.H., Cao Y.C. (2022). Effects of a Combination of Fibrolytic and Amylolytic Enzymes on Ruminal Enzyme Activities, Bacterial Diversity, Blood Profile and Milk Production in Dairy Cows. Animal.

[13] (2018). D.O.F. NMX-Y-098-SCFI-2018. Alimentos Para Animales-Determinación de Humedad en Alimentos Balanceados e Ingredientes Mayores. https://platiica.economia.gob.mx/normalizacion/nmx-y-098-scfi-2018/.

[14] AOAC (2005). Official Method of Analysis.

[15] Vogel K.P., Pedersen J.F., Masterson S.D., Toy J.J. (1999). Evaluation of a Filter Bag System for NDF, ADF, and IVDMD Forage Analysis. Crop Sci..

[16] Thomas M., Serrenho R.C., Puga S.O., Torres J.M., Puga S.O., Stangaferro M. (2023). Effect of Feeding a *Saccharomyces cerevisiae* Fermentation Product to Holstein Cows Exposed to High Temperature and Humidity Conditions on Milk Production Performance and Efficiency—A Pen-Level Trial. J. Dairy Sci..

[17] Colombo E.A., Cooke R.F., Brandão A.P., Wiegand J.B., Schubach K.M., Sowers C.A., Duff G.C., Block E., Gouvêa V.N. (2021). Performance, Health, and Physiological Responses of Newly Received Feedlot Cattle Supplemented with Pre- and Probiotic Ingredients. Animal.

[18] Eslamizad M., Dehghan-Banadaky M., Rezayazdi K., Moradi-Shahrbabak M. (2010). Effects of 6 Times Daily Milking during Early versus Full Lactation of Holstein Cows on Milk Production and Blood Metabolites. J. Dairy Sci..

[19] Sheehy M.R., Mulligan F.J., Taylor S.T., Fahey A.G. (2020). Effects of a Novel Heat-Treated Protein and Carbohydrate Supplement on Feed Consumption, Milk Production, and Cheese Yield in Early-Lactation Dairy Cows. J. Dairy Sci..

[20] Universidad del Valle de Bolivia (2016). Manual de Recolección, Conservación y Envío de Muestras al Laboratorio Para Diagnóstico de Enfermedades Comunes de Los Animales. https://www.woah.org/fileadmin/Home/esp/Animal_Health_in_the_World/docs/pdf/Self-declarations/Archives/Anexo_4._Manual_de_toma_y_remision_de_muestras.pdf.

[21] Villaseñor G.F., Ruvalcaba G.J.M., Espinosa M.M.A., Arteaga G.R.I., Ruvalcaba A.M.J., Montes O.L.R., Hernández J.A.L., Érica A.L. Comparación de Dos Métodos Automatizados Para Análisis de Leche Bovina. Proceedings of the XVII Congreso Internacional de MVZ Especialistas en Bovinos.

[22] (1999). D.O.F. NMX-F-490-1999-NORMEX. Alimentos—Aceites y Grasas—Determinación de La Composición de Ácidos Grasos a Partir de C6 Por Cromatografía de Gases, 1999. https://platiica.economia.gob.mx/normalizacion/nmx-f-490-1999-normex/.

[23] Bolger A.M., Lohse M., Usadel B. (2014). Trimmomatic: A Flexible Trimmer for Illumina Sequence Data. Bioinformatics.

[24] Ewels P.A., Peltzer A., Fillinger S., Patel H., Alneberg J., Wilm A., Garcia M.U., Di Tommaso P., Nahnsen S. (2020). The Nf-Core Framework for Community-Curated Bioinformatics Pipelines. Nat. Biotechnol..

[25] Straub D., Blackwell N., Langarica-Fuentes A., Peltzer A., Nahnsen S., Kleindienst S. (2020). Interpretations of Environmental Microbial Community Studies Are Biased by the Selected 16S RRNA (Gene) Amplicon Sequencing Pipeline. Front. Microbiol..

[26] Callahan B.J., McMurdie P.J., Rosen M.J., Han A.W., Johnson A.J.A., Holmes S.P. (2016). DADA2: High-Resolution Sample Inference from Illumina Amplicon Data. Nat. Methods.

[27] Ewels P., Magnusson M., Lundin S., Käller M. (2016). MultiQC: Summarize Analysis Results for Multiple Tools and Samples in a Single Report. Bioinformatics.

[28] Quast C., Pruesse E., Yilmaz P., Gerken J., Schweer T., Yarza P., Peplies J., Glöckner F.O. (2012). The SILVA Ribosomal RNA Gene Database Project: Improved Data Processing and Web-Based Tools. Nucleic Acids Res..

[29] Caporaso J.G., Kuczynski J., Stombaugh J., Bittinger K., Bushman F.D., Costello E.K., Fierer N., Peña A.G., Goodrich J.K., Gordon J.I. (2010). QIIME Allows Analysis of High-Throughput Community Sequencing Data. Nat. Methods.

[30] Douglas G.M., Maffei V.J., Zaneveld J.R., Yurgel S.N., Brown J.R., Taylor C.M., Huttenhower C., Langille M.G.I. (2020). PICRUSt2 for Prediction of Metagenome Functions. Nat. Biotechnol..

[31] Lu Y., Zhou G., Ewald J., Pang Z., Shiri T., Xia J. (2023). MicrobiomeAnalyst 2.0: Comprehensive Statistical, Functional and Integrative Analysis of Microbiome Data. Nucleic Acids Res..

[32] Silva T.H., Takiya C.S., Vendramini T.H.A., de Jesus E.F., Zanferari F., Rennó F.P. (2016). Effects of Dietary Fibrolytic Enzymes on Chewing Time, Ruminal Fermentation, and Performance of Mid-Lactating Dairy Cows. Anim. Feed Sci. Technol..

[33] Zilio E.M.C., Del Valle T.A., Ghizzi L.G., Takiya C.S., Dias M.S.S., Nunes A.T., Silva G.G., Rennó F.P. (2019). Effects of Exogenous Fibrolytic and Amylolytic Enzymes on Ruminal Fermentation and Performance of Mid-Lactation Dairy Cows. J. Dairy Sci..

[34] Yang J., Refat B., Guevara-Oquendo V.H., Yu P. (2022). Lactational Performance, Feeding Behavior, Ruminal Fermentation and Nutrient Digestibility in Dairy Cows Fed Whole-Plant Faba Bean Silage-Based Diet with Fibrolytic Enzyme. Animal.

[35] Savela M.F.B., Noschang J.P., Barbosa A.A., Feijó J.d.O., Rabassa V.R., Schmitt E., Pino F.A.B.D., Corrêa M.N., Brauner C.C. (2022). Supplementation of a Dried, Fungal Fermentation Product with Fibrolytic Enzymatic Activity in the Diet of Dairy Cows on Feeding Behavior, Metabolic Profile, Milk Yield, and Milk Composition. Livest. Sci..

[36] Peters A., Meyer U., Dänicke S. (2015). Effect of Exogenous Fibrolytic Enzymes on Performance and Blood Profile in Early and Mid-Lactation Holstein Cows. Anim. Nutr..

[37] Kliem K.E., Shingfield K.J. (2016). Manipulation of Milk Fatty Acid Composition in Lactating Cows: Opportunities and Challenges. Eur. J. Lipid Sci. Technol..

[38] Lock A.L., Bauman D.E. (2004). Modifying Milk Fat Composition of Dairy Cows to Enhance Fatty Acids Beneficial to Human Health. Lipids.

[39] Markiewicz-Keszycka M., Czyzak-Runowska G., Lipinska P., Wójtowski J. (2013). Fatty Acid Profile of Milk—A Review. Bull. Vet. Inst. Puławy.

[40] Rojo R., Kholif A.E., Salem A.Z.M., Elghandour M.M.Y., Odongo N.E., Montes De Oca R., Rivero N., Alonso M.U. (2015). Influence of Cellulase Addition to Dairy Goat Diets on Digestion and Fermentation, Milk Production and Fatty Acid Content. J. Agric. Sci..

[41] Shanks O.C., Kelty C.A., Archibeque S., Jenkins M., Newton R.J., McLellan S.L., Huse S.M., Sogin M.L. (2011). Community Structures of Fecal Bacteria in Cattle from Different Animal Feeding Operations. Appl. Environ. Microbiol..

[42] Xue Y., Sun H., Guo H., Nie C., Nan S., Lu Q., Chen C., Zhang W. (2024). Effect of the Supplementation of Exogenous Complex Non-Starch Polysaccharidases on the Growth Performance, Rumen Fermentation and Microflora of Fattening Sheep. Front. Vet. Sci..

[43] Tian Z., Li G., Tang W., Zhu Q., Li X., Du C., Li C., Li J., Zhao C., Zhang L. (2022). Role of Sedum Alfredii and Soil Microbes in the Remediation of Ultra-High Content Heavy Metals Contaminated Soil. Agric. Ecosyst. Environ..

[44] Williamson J.R., Callaway T.R., Lourenco J.M., Ryman V.E. (2022). Characterization of Rumen, Fecal, and Milk Microbiota in Lactating Dairy Cows. Front. Microbiol..

[45] Sun Z., Yu Z., Wang B. (2019). *Perilla frutescens* Leaf Alters the Rumen Microbial Community of Lactating Dairy Cows. Microorganisms.

[46] Wei W., Jiang W., Tian Z., Wu H., Ning H., Yan G., Zhang Z., Li Z., Dong F., Sun Y. (2021). Fecal *g. Streptococcus* and *g. Eubacterium_coprostanoligenes_group* Combined with Sphingosine to Modulate the Serum Dyslipidemia in High-Fat Diet Mice. Clin. Nutr..

[47] Martinez-Fernandez G., Denman S.E., Walker N., Kindermann M., McSweeney C.S. (2024). Programming Rumen Microbiome Development in Calves with the Anti-Methanogenic Compound 3-NOP. Anim. Microbiome.

[48] Adeyemi J.A., Peters S.O., De Donato M., Cervantes A.P., Ogunade I.M. (2020). Effects of a Blend of *Saccharomyces cerevisiae*-Based Direct-Fed Microbial and Fermentation Products on Plasma Carbonyl-Metabolome and Fecal Bacterial Community of Beef Steers. J. Anim. Sci. Biotechnol..

[49] Kabel M.A., Yeoman C.J., Han Y., Dodd D., Abbas C.A., de Bont J.A.M., Morrison M., Cann I.K.O., Mackie R.I. (2011). Biochemical Characterization and Relative Expression Levels of Multiple Carbohydrate Esterases of the Xylanolytic Rumen Bacterium *Prevotella ruminicola* 23 Grown on an Ester-Enriched Substrate. Appl. Environ. Microbiol..

[50] Kim J.N., Méndez–García C., Geier R.R., Iakiviak M., Chang J., Cann I., Mackie R.I. (2017). Metabolic Networks for Nitrogen Utilization in *Prevotella ruminicola* 23. Sci. Rep..

[51] Liu J., Zhang M., Zhang R., Zhu W., Mao S. (2016). Comparative studies of the composition of bacterial microbiota associated with the ruminal content, ruminal epithelium and in the faeces of lactating dairy cows. Microb. Biotechnol..

[52] Castro-Ramos J.J., Solís-Oba A., Solís-Oba M., Calderón-Vázquez C.L., Higuera-Rubio J.M., Castro-Rivera R. (2022). Effect of the Initial PH on the Anaerobic Digestion Process of Dairy Cattle Manure. AMB Express.

[53] Brulin L., Ducrocq S., Estellé J., Even G., Martel S., Merlin S., Audebert C., Croiseau P., Sanchez M.P. (2024). The Fecal Microbiota of Holstein Cows Is Heritable and Genetically Correlated to Dairy Performances. J. Dairy Sci..

[54] Lynch J.B., Gonzalez E.L., Choy K., Faull K.F., Jewell T., Arellano A., Liang J., Yu K.B., Paramo J., Hsiao E.Y. (2023). Gut Microbiota *Turicibacter* Strains Differentially Modify Bile Acids and Host Lipids. Nat. Commun..

[55] Zhang J., Zhang X., Liu H., Wang P., Li L., Bionaz M., Lin P., Yao J. (2024). Altered Bile Acid and Correlations with Gut Microbiome in Transition Dairy Cows with Different Glucose and Lipid Metabolism Status. J. Dairy Sci..

[56] Wang S., Kong F., Liu J., Xia J., Du W., Li S., Wang W. (2023). Comparative Analysis of Rumen Microbiota Composition in Dairy Cows with Simple Indigestion and Healthy Cows. Microorganisms.

[57] Bai H., Lai Z., Zhang J., Zheng X., Zhang J., Jin W., Lin L., Mao S. (2024). Host Genetic Regulation of Specific Functional Groups in the Rumen Microbiome of Dairy Cows: Implications for Lactation Trait. J. Adv. Res..

[58] Guo J., Liu B., Maimai T., Zhao C., Cao Y., Zhang N., Hu X., Fu Y. (2021). Characterization of Bacterial Community in the Rumen of Bovine with Laminitis Using High-Throughput Sequencing. Genes.

[59] Scarsella E., Zecconi A., Cintio M., Stefanon B. (2021). Characterization of Microbiome on Feces, Blood and Milk in Dairy Cows with Different Milk Leucocyte Pattern. Animals.

[60] Duthoit F., Godon J.J., Montel M.C. (2003). Bacterial Community Dynamics during Production of Registered Designation of Origin Salers Cheese as Evaluated by 16S RRNA Gene Single-Strand Conformation Polymorphism Analysis. Appl. Environ. Microbiol..

[61] Colombo M., Castilho N.P.A., Todorov S.D., Nero L.A. (2017). Beneficial and Safety Properties of a *Corynebacterium vitaeruminis* Strain Isolated from the Cow Rumen. Probiotics Antimicrob. Proteins.

[62] Huang S., Ji S., Yan H., Hao Y., Zhang J., Wang Y., Cao Z., Li S. (2020). The Day-to-Day Stability of the Ruminal and Fecal Microbiota in Lactating Dairy Cows. MicrobiologyOpen.

[63] Mu Y., Lin X., Wang Z., Hou Q., Wang Y., Hu Z. (2019). High-Production Dairy Cattle Exhibit Different Rumen and Fecal Bacterial Community and Rumen Metabolite Profile than Low-Production Cattle. MicrobiologyOpen.

[64] Lee H., Fitamo T.M., Nesbø C.L., Guilford N.G.H., Kanger K., Yang M.I., Edwards E.A. (2023). Microbial Community Dynamics of a Sequentially Fed Anaerobic Digester Treating Solid Organic Waste. FEMS Microbiol. Ecol..

[65] Tang M.T., Han H., Yu Z., Tsuruta T., Nishino N. (2017). Variability, Stability, and Resilience of Fecal Microbiota in Dairy Cows Fed Whole Crop Corn Silage. Appl. Microbiol. Biotechnol..

[66] Brulin L., Ducrocq S., Even G., Sanchez M.P., Martel S., Merlin S., Audebert C., Croiseau P., Estellé J. (2024). Short Communication: *Bifidobacterium* Abundance in the Faecal Microbiota Is Strongly Associated with Milk Traits in Dairy Cattle. Animal.

[67] Yang X., Wang J., Cheng J., Zhang D., Huang K., Zhang Y., Li X., Zhao Y., Zhao L., Xu D. (2024). Relationship between Sheep Feces Scores and Gastrointestinal Microorganisms and Their Effects on Growth Traits and Blood Indicators. Front. Microbiol..

[68] Low L., Suleiman K., Shamdas M., Bassilious K., Poonit N., Rossiter A.E., Acharjee A., Loman N., Murray P.I., Wallace G.R. (2022). Gut Dysbiosis in Ocular Mucous Membrane Pemphigoid. Front. Cell Infect. Microbiol..

[69] Zhang L., Piao X. (2022). Different Dietary Protein Sources Influence Growth Performance, Antioxidant Capacity, Immunity, Fecal Microbiota and Metabolites in Weaned Piglets. Anim. Nutr..

[70] Lyu J., Yang Z., Wang E., Liu G., Wang Y., Wang W., Li S. (2022). Possibility of Using By-Products with High NDF Content to Alter the Fecal Short Chain Fatty Acid Profiles, Bacterial Community, and Digestibility of Lactating Dairy Cows. Microorganisms.

[71] Castillo-Lopez E., Haselmann A., Petri R.M., Knaus W., Zebeli Q. (2020). Evaluation of Fecal Fermentation Profile and Bacterial Community in Organically Fed Dairy Cows Consuming Forage-Rich Diets with Different Particle Sizes. J. Dairy Sci..

[72] Falalyeyeva T., Chornenka N., Cherkasova L., Tsyryuk O., Molchek N., Kovalchuk O., Kyriachenko Y., Ostapchenko L., Kobyliak N. (2022). Gut Microbiota Interactions With Obesity. Comprehensive Gut Microbiota.

[73] Bajpai P. (2009). Xylanases. Encyclopedia of Microbiology.

[74] Berrin J.-G., Juge N. (2008). Factors Affecting Xylanase Functionality in the Degradation of Arabinoxylans. Biotechnol. Lett..

[75] Romero J.J., Macias E.G., Ma Z.X., Martins R.M., Staples C.R., Beauchemin K.A., Adesogan A.T. (2016). Improving the Performance of Dairy Cattle with a Xylanase-Rich Exogenous Enzyme Preparation. J. Dairy Sci..

[76] Pech-Cervantes A.A., Ogunade I.M., Jiang Y., Estrada-Reyes Z.M., Arriola K.G., Amaro F.X., Staples C.R., Vyas D., Adesogan A.T. (2021). Effects of a Xylanase-Rich Enzyme on Intake, Milk Production, and Digestibility of Dairy Cows Fed a Diet Containing a High Proportion of Bermudagrass Silage. J. Dairy Sci..

[77] Beauchemin K.A., Holtshausen L. (2010). Developments in Enzyme Usage in Ruminants. Enzymes in Farm Animal Nutrition.

[78] Yang W.Z., Beauchemin K.A., Rode L.M. (2000). A Comparison of Methods of Adding Fibrolytic Enzymes to Lactating Cow Diets. J. Dairy Sci..

