# Supplementary material for: Fecal Microbiota and Performance of Dairy Cattle from a West Mexican Family Dairy Farm Supplemented with a Fiber-Degrading Enzymatic Complex
Source: Vet Sci. 2025 May 25;12(6):518. doi: 10.3390/vetsci12060518 (PMC12197793; doi:10.3390/vetsci12060518)
Supplement: Supplementary file 1 [file vetsci-12-00518-s001.zip › vetsci-3633615-supplementary.pdf]

**SUPPLEMENTARY MATERIAL**

*Article*

**Fecal Microbiota and Performance of Dairy Cattle from a West Mexican Family Dairy Farm Supplemented with a Fiber-Degrading Enzymatic Complex**

**Table S1.** Dry matter intake of cows with and without the inclusion of the enzymatic complex.

| | Group (treatment) | | Significance (*p*-value) | | |
|---|---|---|---|---|---|
| **Week** | **T1 (control)** | **T2 (Hostazym)** | **Treat** | **Wk** | **Treat*wk** |
| | **KgDM/cow/day*** | | | | |
| 2 | 26.84(±0.62) | 26.77(±0.79) | | | |
| 3 | 26.70(±0.95) | 26.08(±1.19) | | | |
| 4 | 27.03(±0.49) | 26.72(±0.13) | | | |
| 5 | 26.82(±0.53) | 27.51(±0.75) | | | |
| 6 | 26.63(±0.57) | 27.52(±0.52) | **<.0001** | 0.0008 | 0.0003 |
| 7 | 25.77(±0.53) | 27.04(±0.41) | | | |
| 8 | 26.26(±0.60) | 27.50(±0.29) | | | |
| 9 | 26.53(±1.05) | 27.68(±0.57) | | | |
| 10 | 26.92(±1.02) | 28.50(±1.04) | | | |

*Mean value ± standard deviation. DMI measured at the pen level. Treat=treatment; wk=week of evaluation; Trat*wk=treatment (group) * week.

**Table S2.** Milk production (total and corrected to 4% fat) of cows with and without the inclusion of the enzymatic complex.

| | Group (treatment) | | Significance (*p*-value) | | |
|---|---|---|---|---|---|
| **Week** | **T1 (control)** | **T2 (Hostazym)** | **Treat** | **Wk** | **Treat*wk** |
| | **MilkKg/cow/day*** | | | | |
| 2 | 38.84(±6.93) | 39.98(±8.28) | | | |
| 4 | 38.36(±7.19) | 39.24(±8.48) | | | |
| 5 | 38.42(±6.68) | 40.44(±8.00) | | | |
| 6 | 36.75(±7.32) | 39.27(±6.46) | **0.007** | 0.305 | 0.981 |
| 7 | 35.59(±7.67) | 38.59(±6.46) | | | |
| 8 | 34.62(±9.68) | 37.78(±6.77) | | | |
| 9 | 36.41(±7.26) | 38.15(±7.44) | | | |
| 10 | 34.57(±7.08) | 38.63(±6.79) | | | |
| | **CM4%fat/cow/day*** | | | | |
| 2 | 33.49(±5.97) | 34.85(±7.22) | | | |
| 4 | 33.92(±6.18) | 34.40(±7.44) | | | |
| 5 | 33.36(±5.80) | 35.94(±7.11) | | | |
| 6 | 32.39(±6.45) | 34.53(±5.68) | **0.039** | 0.519 | 0.975 |
| 7 | 31.56(±6.80) | 33.98(±5.68) | | | |
| 8 | 31.08(±8.69) | 33.10(±5.93) | | | |
| 9 | 32.88(±4.88) | 32.67(±6.37) | | | |
| 10 | 31.66(±6.49) | 33.39(±5.87) | | | |

*Mean value ± standard deviation. Treat=treatment; wk=week of evaluation; Trat*wk=treatment (group) * week.

**Table S3.** Physicochemical composition profile of milk produced by cows with and without the inclusion of the enzymatic complex.

| | Group (treatment) | | Significance (*p*-value) | | |
|---|---|---|---|---|---|
| **Week** | **T1 (control)** | **T2 (Hostazym)** | **Treat** | **Wk** | **Treat*wk** |
| | | **Fat (%)*** | | | |
| 2 | 3.08(±0.10) | 3.14(±0.05) | 0.2260 | 0.9809 | 0.6703 |
| 4 | 3.23(±0.34) | 3.18(±0.01) | | | |
| 5 | 3.12(±0.33) | 3.26(±0.22) | | | |
| 6 | 3.21(±0.39) | 3.20(±0.10) | | | |
| 7 | 3.25(±0.45) | 3.20(±0.12) | | | |
| 8 | 3.32(±0.42) | 3.17(±0.08) | | | |
| 9 | 3.35(±0.24) | 3.04(±0.07) | | | |
| 10 | 3.44(±0.25) | 3.10(±0.02) | | | |
| | | **Density (Kg/L)*** | | | |
| 2 | 1028.58(±0.39) | 1028.08(±0.22) | **0.0005** | **<.0001** | **<.0001** |
| 4 | 1028.66(±0.52) | 1028.10(±0.24) | | | |
| 5 | 1028.75(±0.60) | 1028.10(±0.24) | | | |
| 6 | 1028.83(±0.66) | 1028.11(±0.25) | | | |
| 7 | 1028.91(±0.70) | 1028.11(±0.25) | | | |
| 8 | 1029.00(±0.73) | 1028.12(±0.26) | | | |
| 9 | 1029.04(±0.70) | 1028.15(±0.28) | | | |
| 10 | 1029.09(±0.67) | 1028.20(±0.29) | | | |
| | | **Lactose (%)*** | | | |
| 2 | 4.80(±0.08) | 4.68(±0.02) | **<.0001** | **<.0001** | **<.0001** |
| 4 | 4.75(±0.04) | 4.63(±0.07) | | | |
| 5 | 4.76(±0.03) | 4.76(±0.03) | | | |
| 6 | 4.79(±0.03) | 4.61(±0.04) | | | |
| 7 | 4.85(±0.09) | 4.85(±0.15) | | | |
| 8 | 4.85(±0.09) | 4.68(±0.06) | | | |
| 9 | 4.79(±0.05) | 4.80(±0.04) | | | |
| 10 | 4.77(±0.03) | 4.71(±0.02) | | | |
| | | **Non-fat solids (%)*** | | | |
| 2 | 8.70(±0.09) | 8.47(±0.04) | **<.0001** | **<.0001** | **<.0001** |
| 4 | 8.58(±0.07) | 8.37(±0.13) | | | |
| 5 | 8.61(±0.06) | 8.61(±0.06) | | | |
| 6 | 8.66(±0.05) | 8.34(±0.06) | | | |
| 7 | 8.78(±0.17) | 8.77(±0.28) | | | |
| 8 | 8.78(±0.17) | 8.46(±0.12) | | | |
| 9 | 8.65(±0.09) | 8.68(±0.08) | | | |
| 10 | 8.63(±0.05) | 8.52(±0.04) | | | |
| | | **Protein (%)*** | | | |
| 2 | 3.10(±0.03) | 3.01(±0.02) | **<.0001** | **<.0001** | **<.0001** |
| 4 | 3.05(±0.02) | 2.98(±0.05) | | | |
| 5 | 3.06(±0.02) | 3.06(±0.02) | | | |
| 6 | 3.08(±0.02) | 2.96(±0.02) | | | |
| 7 | 3.13(±0.06) | 3.12(±0.10) | | | |
| 8 | 3.13(±0.06) | 3.01(±0.04) | | | |

| | | | | | |
|---|---|---|---|---|---|
| 9 | 3.08(±0.03) | 3.09(±0.03) | | | |
| 10 | 3.07(±0.02) | 3.03(±0.01) | | | |

*Mean value ± standard deviation. Treat=treatment; wk=week of evaluation; Trat*wk=treatment (group) * week.

**Table S4.** Fatty acid profile in milk produced by cows with and without the inclusion of the enzymatic complex.

| | Group (treatment) | | Significance *p*-value) | | |
|---|---|---|---|---|---|
| **Week** | **T1 (control)** | **T2 (Hostazym)** | **Treat** | **Wk** | **Treat*wk** |
| | | | **Palmitic acid (g/L)** | | |
| 2 | 11.41(±0.10) | 12.69(±0.01) | | | |
| 4 | 12.08(±0.32) | 13.15(±1.45) | | | |
| 5 | 11.74(±0.36) | 12.46(±0.18) | | | |
| 6 | 11.86(±0.03) | 12.55(±0.69) | **0.029** | 0.474 | 0.335 |
| 8 | 11.91(±0.62) | 12.19(±0.26) | | | |
| 10 | 12.92(±0.06) | 12.37(±0.14) | | | |
| | | | **Estearic acid (g/L)** | | |
| 2 | 3.53(±0.16) | 3.56(±0.07) | | | |
| 4 | 3.60(±0.26) | 2.07(±2.31) | | | |
| 5 | 3.18(±0.21) | 3.52(±0.04) | | | |
| 6 | 3.45(±0.13) | 3.57(±0.16) | 0.436 | 0.490 | 0.481 |
| 8 | 3.88(±0.18) | 3.70(±0.10) | | | |
| 10 | 3.68(±0.01) | 3.54(±0.11) | | | |
| | | | **Oleic acid (g/L)** | | |
| 2 | 8.12(±0.21) | 7.92(±0.62) | | | |
| 4 | 8.10(±0.18) | 9.63(±0.66) | | | |
| 5 | 7.68(±0.04) | 8.50(±1.11) | | | |
| 6 | 7.91(±0.04) | 8.67(±0.15) | 0.065 | 0.145 | **0.015** |
| 8 | 8.05(±0.16) | 8.36(±0.13) | | | |
| 10 | 8.97(±0.01) | 7.89(±0.09) | | | |
| | | | **Linoleic acid (g/L)** | | |
| 2 | 0.90(±0.08) | 0.91(±0.05) | | | |
| 4 | 0.90(±0.04) | 0.99(±0.13) | | | |
| 5 | 0.93(±0.04) | 0.88(±0.01) | | | |
| 6 | 0.89(±0.03) | 0.90(±0.08) | 0.949 | 0.721 | 0.609 |
| 8 | 0.94(±0.10) | 0.95(±0.02) | | | |
| 10 | 0.98(±0.04) | 0.92(±0.01) | | | |
| | | | **Linolenic acid (g/L)** | | |
| 2 | 0.15(±0.01) | 0.15(±0.01) | | | |
| 4 | 0.17(±0.01) | 0.17(±0.03) | | | |
| 5 | 0.16(±0.01) | 0.15(±0.01) | | | |
| 6 | 0.16(±0.01) | 0.17(±0.01) | 0.721 | **0.024** | 0.451 |
| 8 | 0.16(±0.01) | 0.17(±0.01) | | | |
| 10 | 0.19(±0.01) | 0.17(±0.01) | | | |
| | | | **Saturated fat (g/L)** | | |

| | | | | | |
|---|---|---|---|---|---|
| 2 | 9.43(±7.66) | 16.26(±0.08) | | | |
| 4 | 15.67(±0.58) | 15.20(±0.86) | | | |
| 5 | 14.91(±0.57) | 15.98(±0.23) | | | |
| 6 | 15.31(±0.11) | 16.12(±0.85) | 0.193 | 0.383 | 0.251 |
| 8 | 15.79(±0.80) | 15.88(±0.35) | | | |
| 10 | 16.59(±0.04) | 15.91(±0.03) | | | |
| | | **Monounsaturated fat (g/L)** | | | |
| 2 | 8.12(±0.21) | 7.92(±0.62) | | | |
| 4 | 8.10(±0.18) | 9.63(±0.66) | | | |
| 5 | 7.68(±0.04) | 8.50(±1.11) | | | |
| 6 | 7.91(±0.04) | 8.67(±0.15) | 0.065 | 0.145 | **0.015** |
| 8 | 8.05(±0.16) | 8.36(±0.13) | | | |
| 10 | 8.97(±0.01) | 7.89(±0.09) | | | |
| | | **Polyunsaturated fat (g/L)** | | | |
| 2 | 1.05(±0.09) | 1.05(±0.06) | | | |
| 4 | 1.06(±0.03) | 1.16(±0.16) | | | |
| 5 | 1.08(±0.04) | 1.03(±0.02) | | | |
| 6 | 1.05(±0.04) | 1.07(±0.09) | 0.978 | 0.529 | 0.567 |
| 8 | 1.10(±0.11) | 1.11(±0.03) | | | |
| 10 | 1.17(±0.04) | 1.08(±0.01) | | | |

*Mean value ± standard deviation. Treat=treatment; wk=week of evaluation; Trat*wk=treatment (group) * week.

**Table S5.** Main observed Phyla in fecal bacterial populations from feces of cows with and without the inclusion of the enzymatic complex.

| | Average relative abundance (Group_time) | | | | | | Significance |
|---|---|---|---|---|---|---|---|
| **Phylum** | **TC_T1** | **TE_T1** | **TC_T2** | **TE_T2** | **TC_T3** | **TE_T3** | ***p*-value*** |
| Firmicutes | 65.05 | 66.29 | 55.56 | 70.12 | 69.19 | 65.20 | 0.560 |
| Bacteroidota | 15.97 | 14.36 | 26.73 | 16.85 | 20.17 | 19.96 | 0.791 |
| Actinobacteriota | 14.62 | 14.36 | 12.23 | 9.39 | 7.44 | 11.19 | 0.874 |
| Proteobacteria | 0.33 | 0.76 | 0.87 | 0.15 | 0.70 | 0.52 | 0.671 |
| Spirochaetota | 0.15 | 0.24 | 0.40 | 0.20 | 0.28 | 0.21 | 0.711 |
| Patescibacteria | 1.05 | 1.29 | 1.06 | 1.09 | 1.09 | 1.33 | **0.011** |
| Cyanobacteria | 0.02 | 0.08 | 0.00 | 0.02 | 0.03 | 0.04 | 0.282 |
| Other | 2.82 | 2.62 | 3.16 | 2.18 | 1.10 | 1.55 | 0.874 |

* Calculated thought the Kruskal-Wallis pair comparison test at significance level of 0.05.

**Table S6.** Relative abundance of the families of bacteria observed in the fecal microbiota of cows supplemented with the enzymatic complex (TE) and without supplementation (TC) at different sampling times (T1=week 1, T2=week 5, T3=week 10).

| | Average relative abundance (Group_time) | Significance |
|---|---|---|

| Family | TC_T1 | TE_T1 | TC_T2 | TE_T2 | TC_T3 | TE_T3 | *p*-value* |
|---|---|---|---|---|---|---|---|
| Lachnospiraceae | 16.27 | 16.66 | 12.46 | 16.44 | 15.48 | 17.91 | 0.223 |
| Peptostreptococcaceae | 13.10 | 11.75 | 10.59 | 14.45 | 13.99 | 11.78 | 0.560 |
| Erysipelotrichaceae | 10.73 | 9.59 | 7.88 | 8.13 | 11.31 | 6.44 | **0.050** |
| Bifidobacteriaceae | 8.34 | 6.14 | 10.04 | 4.36 | 4.51 | 7.29 | 0.368 |
| Rikenellaceae | 7.57 | 5.54 | 14.22 | 8.89 | 10.83 | 10.39 | 0.491 |
| Anaerovoracaceae | 5.40 | 4.68 | 2.62 | 6.98 | 6.45 | 7.24 | 0.368 |
| Clostridiaceae | 5.35 | 5.08 | 3.74 | 6.03 | 5.78 | 3.84 | 0.634 |
| Atopobiaceae | 4.27 | 5.87 | 1.40 | 3.13 | 1.45 | 2.39 | 0.315 |
| Oscillospiraceae | 3.48 | 3.28 | 5.58 | 4.28 | 3.60 | 4.17 | 0.874 |
| Other_Clostridia | 3.42 | 3.49 | 2.75 | 4.16 | 3.26 | 3.32 | 0.153 |
| Other_Bacteroidales | 2.37 | 2.38 | 3.09 | 2.33 | 2.23 | 3.12 | 0.491 |
| Bacteroidaceae | 1.98 | 1.41 | 2.55 | 1.52 | 1.45 | 1.18 | 0.138 |
| Other_Bacteroidia | 1.64 | 2.04 | 1.42 | 1.14 | 0.93 | 1.08 | 0.958 |
| Other_Actinobacteriota | 1.59 | 1.29 | 0.56 | 1.11 | 0.80 | 0.86 | 0.711 |
| Christensenellaceae | 1.54 | 1.78 | 1.57 | 1.95 | 1.74 | 2.19 | 0.080 |
| Other_Bacilli_RF39 | 1.50 | 1.43 | 1.30 | 1.25 | 1.47 | 1.57 | 0.832 |
| Other_Firmicutes | 1.35 | 1.39 | 1.71 | 1.29 | 1.21 | 1.27 | 0.711 |
| Saccharimonadaceae | 1.05 | 1.29 | 1.06 | 1.09 | 1.08 | 1.33 | **0.011** |
| Prevotellaceae | 1.04 | 1.64 | 2.57 | 1.39 | 1.84 | 2.21 | 0.791 |
| Other_Oscillospirales | 0.89 | 0.81 | 0.81 | 1.13 | 0.33 | 0.80 | 0.711 |
| [Eubacterium] coprostanoligenes group | 0.62 | 1.03 | 0.95 | 1.11 | 0.77 | 1.50 | **0.017** |
| Paludibacteraceae | 0.39 | 0.16 | 0.55 | 0.00 | 0.00 | 0.00 | 0.313 |
| Muribaculaceae | 0.37 | 0.40 | 0.99 | 0.56 | 1.45 | 0.94 | 0.427 |
| Ruminococcaceae | 0.33 | 0.33 | 1.21 | 0.34 | 0.40 | 0.65 | 0.874 |
| Other_Clostridia UCG-014 | 0.23 | 0.27 | 0.24 | 0.43 | 0.40 | 0.33 | 0.368 |
| Bacteroidales RF16 group | 0.23 | 0.45 | 1.00 | 0.53 | 0.59 | 0.62 | 0.874 |
| Pasteurellaceae | 0.21 | 0.38 | 0.61 | 0.00 | 0.02 | 0.00 | 0.484 |
| Marinifilaceae | 0.20 | 0.02 | 0.13 | 0.19 | 0.13 | 0.17 | 0.396 |
| Monoglobaceae | 0.17 | 0.22 | 0.17 | 0.13 | 0.04 | 0.20 | 0.312 |
| Spirochaetaceae | 0.15 | 0.24 | 0.40 | 0.20 | 0.28 | 0.21 | 0.711 |
| Eggerthellaceae | 0.14 | 0.47 | 0.13 | 0.43 | 0.52 | 0.44 | 0.266 |
| p-2534-18B5 gut group | 0.12 | 0.19 | 0.06 | 0.16 | 0.18 | 0.11 | 1.000 |
| Other_Coriobacteriales | 0.11 | 0.17 | 0.03 | 0.19 | 0.10 | 0.11 | 0.124 |
| Other_Peptostreptococcales-Tissierellales | 0.11 | 1.50 | 0.17 | 0.35 | 0.81 | 0.34 | 0.223 |
| Enterobacteriaceae | 0.09 | 0.09 | 0.19 | 0.12 | 0.22 | 0.18 | 0.289 |
| Lactobacillaceae | 0.09 | 0.04 | 0.02 | 0.00 | 0.01 | 0.04 | 0.587 |
| Other_Erysipelotrichales | 0.08 | 0.07 | 0.00 | 0.83 | 0.70 | 0.55 | 0.337 |
| Coriobacteriaceae | 0.08 | 0.06 | 0.07 | 0.02 | 0.01 | 0.04 | 0.747 |
| Other_Bacilli | 0.07 | 0.65 | 0.66 | 0.07 | 0.36 | 0.12 | 0.525 |
| Other_Clostridia vadinBB60 group | 0.06 | 0.11 | 0.25 | 0.16 | 0.15 | 0.14 | 0.791 |

| | | | | | | | |
|---|---|---|---|---|---|---|---|
| Erysipelatoclostridiaceae | 0.06 | 0.13 | 0.14 | 0.04 | 0.03 | 0.09 | 0.457 |
| Oscillospirales_UCG-010 | 0.04 | 0.14 | 0.45 | 0.33 | 0.38 | 0.41 | 0.457 |
| Barnesiellaceae | 0.03 | 0.07 | 0.13 | 0.12 | 0.14 | 0.12 | 0.522 |
| Bacillaceae | 0.03 | 0.23 | 0.05 | 0.08 | 0.00 | 0.00 | 0.336 |
| Butyricicoccaceae | 0.02 | 0.10 | 0.08 | 0.05 | 0.10 | 0.15 | 0.197 |
| Hungateiclostridiaceae | 0.01 | 0.02 | 0.08 | 0.06 | 0.02 | 0.02 | 0.585 |
| Succinivibrionaceae | 0.01 | 0.03 | 0.01 | 0.02 | 0.00 | 0.03 | 0.161 |
| Paenibacillaceae | 0.01 | 0.02 | 0.00 | 0.00 | 0.01 | 0.00 | 0.949 |
| Other_Actinobacteria | 0.01 | 0.05 | 0.00 | 0.00 | 0.02 | 0.00 | 0.237 |
| Tannerellaceae | 0.01 | 0.01 | 0.00 | 0.00 | 0.00 | 0.00 | 0.854 |
| Other_Kiritimatiellae_WCHB1-41 | 0.01 | 0.00 | 0.00 | 0.00 | 0.00 | 0.00 | 0.783 |
| Moraxellaceae | 0.01 | 0.13 | 0.00 | 0.01 | 0.43 | 0.04 | 0.907 |
| Planococcaceae | 0.00 | 1.19 | 0.01 | 0.01 | 0.00 | 0.00 | 0.415 |
| Brevibacillaceae | 0.00 | 0.01 | 0.00 | 0.00 | 0.00 | 0.00 | 0.641 |
| Pirellulaceae | 0.00 | 0.01 | 0.00 | 0.00 | 0.00 | 0.00 | 0.745 |
| Fibrobacteraceae | 0.00 | 0.01 | 0.00 | 0.00 | 0.00 | 0.00 | 0.378 |
| Enterococcaceae | 0.00 | 0.00 | 0.00 | 0.00 | 0.24 | 0.02 | 0.221 |
| Corynebacteriaceae | 0.00 | 0.15 | 0.01 | 0.02 | 0.02 | 0.06 | **0.027** |
| Bacteroidales_F082 | 0.00 | 0.03 | 0.00 | 0.01 | 0.02 | 0.01 | 0.531 |
| Other_Izemoplasmatales | 0.00 | 0.01 | 0.00 | 0.00 | 0.05 | 0.05 | 0.953 |
| Pseudomonadaceae | 0.00 | 0.00 | 0.00 | 0.00 | 0.00 | 0.00 | 1.000 |
| Listeriaceae | 0.00 | 0.00 | 0.00 | 0.00 | 0.00 | 0.00 | 1.000 |
| Staphylococcaceae | 0.00 | 0.00 | 0.00 | 0.00 | 0.00 | 0.00 | 1.000 |
| Oscillospirales_UCG-011 | 0.00 | 0.00 | 0.00 | 0.03 | 0.04 | 0.05 | 0.350 |
| Akkermansiaceae | 0.00 | 0.01 | 0.00 | 0.03 | 0.00 | 0.02 | **0.024** |
| Burkholderiaceae | 0.00 | 0.00 | 0.00 | 0.00 | 0.03 | 0.09 | 0.587 |
| Chitinophagaceae | 0.00 | 0.00 | 0.00 | 0.00 | 0.39 | 0.00 | 0.257 |
| Other_Enterobacterales | 0.00 | 0.06 | 0.00 | 0.00 | 0.00 | 0.18 | 0.198 |
| Other | 3.04 | 3.20 | 3.32 | 2.31 | 1.22 | 1.62 | 0.958 |

* Calculated thought the Kruskal-Wallis pair comparison test at significance level of 0.05.

**Table S7.** Relative abundance of the families of bacteria observed in the fecal microbiota of cows supplemented with the enzymatic complex (TE) and without supplementation (TC) at different sampling times (T1=week 1, T2=week 5, T3=week 10).

| | **Average relative abundance (Group time)** | | | | | | **Significance** |
|---|---|---|---|---|---|---|---|
| ***Genus*** | **TC_T1** | **TC_T2** | **TC_T3** | **TE_T1** | **TE_T2** | **TE_T3** | ***p*-value*** |
| *Lachnospiraceae_NK3A20_group* | 8.754 | 6.436 | 9.401 | 8.692 | 9.555 | 9.875 | 0.491 |
| *Bifidobacterium* | 8.281 | 10.028 | 4.511 | 6.057 | 4.358 | 7.283 | 0.266 |
| *Turicibacter* | 7.811 | 7.589 | 10.780 | 5.513 | 7.269 | 5.649 | **0.017** |
| *Rikenellaceae_RC9_gut_group* | 5.801 | 8.929 | 7.591 | 3.653 | 6.529 | 7.524 | 0.315 |
| *Paeniclostridium* | 4.314 | 4.038 | 6.724 | 4.030 | 6.581 | 4.890 | 0.791 |

| | | | | | | | |
|---|---|---|---|---|---|---|---|
| *Other_Peptostreptococcaceae* | 4.003 | 2.277 | 1.561 | 1.975 | 1.930 | 1.844 | 0.223 |
| *Mogibacterium* | 3.605 | 1.878 | 4.239 | 2.702 | 4.386 | 4.584 | 0.634 |
| *Other_Lachnospiraceae* | 3.455 | 2.800 | 2.348 | 4.031 | 3.446 | 3.422 | **0.030** |
| *Other_Clostridia* | 3.421 | 2.752 | 3.265 | 3.489 | 4.163 | 3.318 | 0.153 |
| *Olsenella* | 3.414 | 1.172 | 1.219 | 5.115 | 2.784 | 2.160 | 0.368 |
| *Romboutsia* | 3.413 | 3.291 | 4.750 | 4.492 | 4.843 | 3.852 | 0.711 |
| *Other_Clostridiaceae* | 2.822 | 1.157 | 1.190 | 1.658 | 1.886 | 1.152 | 0.791 |
| *Other_1* | 2.794 | 3.142 | 1.085 | 2.591 | 2.140 | 1.522 | 0.791 |
| *Catenisphaera* | 2.634 | 0.124 | 0.408 | 3.725 | 0.706 | 0.569 | 0.874 |
| *Clostridium_sensu_stricto_1* | 2.467 | 2.579 | 4.584 | 3.399 | 4.141 | 2.672 | 0.958 |
| *Oscillospiraceae_UCG_005* | 2.390 | 3.228 | 2.846 | 2.200 | 3.171 | 2.931 | 0.634 |
| *Other_Bacteroidales* | 2.375 | 3.092 | 2.227 | 2.377 | 2.331 | 3.120 | 0.491 |
| *Bacteroides* | 1.977 | 2.555 | 1.449 | 1.414 | 1.523 | 1.176 | 0.153 |
| *Other_Bacteroidia* | 1.644 | 1.416 | 0.932 | 2.036 | 1.144 | 1.084 | 0.958 |
| *Other_Actinobacteriota* | 1.591 | 0.559 | 0.799 | 1.286 | 1.111 | 0.860 | 0.711 |
| *Christensenellaceae_R_7_group* | 1.522 | 1.572 | 1.629 | 1.776 | 1.854 | 2.149 | 0.064 |
| *Other_Bacilli_RF39* | 1.499 | 1.304 | 1.469 | 1.434 | 1.248 | 1.568 | 0.791 |
| *Family_XIII_AD3011_group* | 1.471 | 0.652 | 1.512 | 1.340 | 1.628 | 1.668 | 0.315 |
| *Cellulosilyticum* | 1.432 | 0.851 | 1.557 | 0.721 | 0.667 | 1.101 | 0.223 |
| *Alistipes* | 1.424 | 4.072 | 2.076 | 1.653 | 1.788 | 2.057 | 0.874 |
| *Other_Firmicutes* | 1.346 | 1.707 | 1.208 | 1.390 | 1.289 | 1.274 | 0.711 |
| *Paraclostridium* | 1.178 | 0.848 | 0.733 | 0.761 | 0.839 | 0.844 | 0.315 |
| *Candidatus_Saccharimonas* | 1.049 | 1.057 | 1.078 | 1.289 | 1.094 | 1.327 | **0.010** |
| *Other_Oscillospirales* | 0.889 | 0.810 | 0.334 | 0.805 | 1.127 | 0.796 | 0.711 |
| *Acetitomaculum* | 0.838 | 0.523 | 1.128 | 0.697 | 0.910 | 1.023 | 0.874 |
| *Other_Oscillospiraceae* | 0.785 | 1.872 | 0.135 | 0.737 | 0.740 | 0.947 | 0.314 |
| *Other_Atopobiaceae* | 0.701 | 0.039 | 0.181 | 0.459 | 0.156 | 0.128 | 0.711 |
| *Other_Eubacterium_coprostanoligenes_group* | 0.618 | 0.948 | 0.770 | 1.031 | 1.109 | 1.504 | **0.017** |
| *Ruminococcus_gauvreauii_group* | 0.517 | 0.441 | 0.300 | 0.581 | 0.299 | 0.823 | 0.223 |
| *Prevotellaceae_UCG_004* | 0.394 | 0.720 | 0.650 | 0.446 | 0.606 | 0.670 | 0.491 |
| *Paludibacteraceae_F0058* | 0.394 | 0.528 | 0.000 | 0.161 | 0.000 | 0.000 | 0.313 |
| *Other_Muribaculaceae* | 0.366 | 0.989 | 1.448 | 0.403 | 0.560 | 0.936 | 0.427 |
| *Other_Rikenellaceae* | 0.346 | 1.012 | 1.143 | 0.148 | 0.531 | 0.719 | 0.289 |
| *Dorea* | 0.332 | 0.000 | 0.000 | 0.245 | 0.084 | 0.022 | 0.334 |
| *Ruminococcus_gnavus_group* | 0.284 | 0.122 | 0.172 | 0.215 | 0.309 | 0.056 | 0.368 |
| *Prevotellaceae_UCG_001* | 0.247 | 0.375 | 0.246 | 0.272 | 0.141 | 0.312 | 0.368 |
| *Oscillospira* | 0.235 | 0.421 | 0.446 | 0.205 | 0.359 | 0.255 | 0.455 |
| *Other_Clostridia_UCG_014* | 0.228 | 0.241 | 0.398 | 0.272 | 0.430 | 0.330 | 0.368 |
| *Other_Bacteroidales_RF16_group* | 0.226 | 0.997 | 0.590 | 0.449 | 0.534 | 0.621 | 0.874 |
| *Other_Ruminococcaceae* | 0.223 | 0.431 | 0.236 | 0.178 | 0.198 | 0.510 | 0.958 |
| *Histophilus* | 0.208 | 0.613 | 0.015 | 0.383 | 0.000 | 0.000 | 0.448 |
| *Odoribacter* | 0.199 | 0.129 | 0.127 | 0.017 | 0.191 | 0.167 | 0.427 |
| *Clostridioides* | 0.194 | 0.135 | 0.153 | 0.495 | 0.261 | 0.345 | **0.017** |
| *Eubacterium_brachy_group* | 0.194 | 0.000 | 0.047 | 0.151 | 0.038 | 0.150 | 0.265 |

| | | | | | | | |
|---|---|---|---|---|---|---|---|
| *Prevotellaceae_UCG_003* | 0.189 | 0.520 | 0.405 | 0.454 | 0.504 | 0.678 | **0.039** |
| *Eubacterium_eligens_group* | 0.187 | 0.000 | 0.213 | 0.079 | 0.198 | 0.275 | 0.670 |
| *Catonella* | 0.172 | 0.058 | 0.045 | 0.114 | 0.178 | 0.104 | 0.426 |
| *Monoglobus* | 0.171 | 0.172 | 0.044 | 0.221 | 0.127 | 0.196 | 0.315 |
| *Other_Prevotellaceae* | 0.166 | 0.495 | 0.318 | 0.258 | 0.091 | 0.216 | 0.265 |
| *Treponema* | 0.147 | 0.389 | 0.281 | 0.216 | 0.168 | 0.197 | 0.560 |
| *Libanicoccus* | 0.135 | 0.000 | 0.000 | 0.099 | 0.000 | 0.000 | 0.783 |
| *Howardella* | 0.131 | 0.063 | 0.054 | 0.252 | 0.085 | 0.125 | 0.186 |
| *Other_Bacteroidales_p_2534_18B5_gut_group* | 0.125 | 0.061 | 0.178 | 0.186 | 0.159 | 0.105 | 0.958 |
| *Other_Eggerthellaceae* | 0.122 | 0.133 | 0.476 | 0.345 | 0.381 | 0.409 | 0.266 |
| *Other_Coriobacteriales* | 0.113 | 0.029 | 0.097 | 0.170 | 0.190 | 0.110 | 0.101 |
| *Ohter_Peptostreptococcales_Tissierellales* | 0.110 | 0.167 | 0.811 | 1.499 | 0.346 | 0.335 | 0.223 |
| *Incertae_Sedis* | 0.084 | 0.336 | 0.008 | 0.074 | 0.042 | 0.016 | 0.913 |
| *Other_Anaerovoracaceae* | 0.081 | 0.072 | 0.631 | 0.418 | 0.901 | 0.637 | 0.101 |
| *Salmonella* | 0.080 | 0.138 | 0.000 | 0.058 | 0.027 | 0.031 | 0.480 |
| *Other_Erysipelotrichales* | 0.080 | 0.000 | 0.704 | 0.073 | 0.833 | 0.554 | 0.337 |
| *Collinsella* | 0.079 | 0.065 | 0.010 | 0.064 | 0.022 | 0.037 | 0.709 |
| *Anaerorhabdus_furcosa_group* | 0.076 | 0.097 | 0.030 | 0.077 | 0.035 | 0.031 | 0.709 |
| *Other_Bacilli__* | 0.074 | 0.659 | 0.359 | 0.654 | 0.070 | 0.124 | 0.491 |
| *Syntrophococcus* | 0.071 | 0.106 | 0.013 | 0.320 | 0.328 | 0.205 | **0.005** |
| *Other_Lactobacillales* | 0.069 | 0.000 | 0.050 | 0.048 | 0.000 | 0.000 | 0.313 |
| *Erysipelotrichaceae_UCG_007* | 0.066 | 0.000 | 0.032 | 0.051 | 0.056 | 0.000 | 0.521 |
| *Other_Erysipelotrichaceae* | 0.064 | 0.007 | 0.000 | 0.069 | 0.015 | 0.082 | 0.096 |
| *Other_Clostridia_vadinBB60_group* | 0.062 | 0.252 | 0.149 | 0.115 | 0.159 | 0.138 | 0.791 |
| *Sarcina* | 0.058 | 0.000 | 0.000 | 0.026 | 0.000 | 0.000 | 0.578 |
| *Raoultibacter* | 0.055 | 0.000 | 0.000 | 0.000 | 0.000 | 0.000 | 0.098 |
| *Ligilactobacillus* | 0.050 | 0.000 | 0.005 | 0.027 | 0.004 | 0.014 | 0.204 |
| *Eubacterium_hallii_group* | 0.046 | 0.026 | 0.140 | 0.172 | 0.124 | 0.197 | 0.100 |
| *Eubacterium_nodatum_group* | 0.045 | 0.015 | 0.019 | 0.068 | 0.030 | 0.178 | 0.166 |
| *Prevotella* | 0.042 | 0.294 | 0.184 | 0.164 | 0.037 | 0.236 | 1.000 |
| *Blautia* | 0.041 | 0.000 | 0.000 | 0.026 | 0.024 | 0.000 | 0.816 |
| *Other_Oscillospirales_UCG_010* | 0.040 | 0.449 | 0.382 | 0.139 | 0.333 | 0.407 | 0.426 |
| *Pseudoscardovia* | 0.035 | 0.000 | 0.000 | 0.059 | 0.000 | 0.002 | 0.949 |
| *Oscillospiraceae_NK4A214_group* | 0.034 | 0.000 | 0.018 | 0.048 | 0.008 | 0.035 | 0.611 |
| *Other_Barnesiellaceae* | 0.031 | 0.131 | 0.130 | 0.064 | 0.118 | 0.110 | 0.711 |
| *Other_Coriobacteriia* | 0.028 | 0.000 | 0.000 | 0.045 | 0.000 | 0.002 | 0.334 |
| *Bacillus* | 0.027 | 0.051 | 0.000 | 0.215 | 0.077 | 0.000 | 0.337 |
| *Erysipelotrichaceae_UCG_002* | 0.027 | 0.000 | 0.000 | 0.078 | 0.010 | 0.068 | 0.054 |
| *Streptococcus* | 0.026 | 0.041 | 0.000 | 0.015 | 0.000 | 0.001 | 0.429 |
| *Other_Lactobacillaceae* | 0.025 | 0.000 | 0.000 | 0.005 | 0.000 | 0.000 | 0.783 |
| *Atopobium* | 0.024 | 0.186 | 0.047 | 0.064 | 0.116 | 0.090 | 0.313 |
| *Dielma* | 0.024 | 0.042 | 0.000 | 0.010 | 0.000 | 0.002 | 0.847 |
| *Oscillospiraceae_UCG_002* | 0.022 | 0.056 | 0.006 | 0.061 | 0.000 | 0.000 | 0.403 |
| *Solobacterium* | 0.022 | 0.000 | 0.000 | 0.000 | 0.000 | 0.000 | 0.257 |

| | | | | | | | |
|---|---|---|---|---|---|---|---|
| *Other_Gastranaerophilales* | 0.020 | 0.000 | 0.028 | 0.084 | 0.022 | 0.040 | 0.309 |
| *Other_Christensenellaceae* | 0.020 | 0.000 | 0.113 | 0.000 | 0.094 | 0.041 | 0.953 |
| *Other_Bifidobacteriaceae* | 0.019 | 0.008 | 0.000 | 0.012 | 0.000 | 0.004 | 0.448 |
| *Denitrobacterium* | 0.017 | 0.000 | 0.000 | 0.016 | 0.000 | 0.000 | 0.816 |
| *Kandleria* | 0.015 | 0.000 | 0.000 | 0.000 | 0.000 | 0.000 | 0.257 |
| *UCG_009* | 0.015 | 0.077 | 0.044 | 0.041 | 0.017 | 0.075 | 0.664 |
| *Arcicella* | 0.015 | 0.000 | 0.000 | 0.000 | 0.000 | 0.020 | 0.927 |
| *Faecalitalea* | 0.014 | 0.022 | 0.020 | 0.018 | 0.002 | 0.037 | 0.704 |
| *Shuttleworthia* | 0.014 | 0.010 | 0.049 | 0.016 | 0.121 | 0.027 | 0.394 |
| *Desulfovibrio* | 0.014 | 0.000 | 0.001 | 0.000 | 0.000 | 0.001 | 0.313 |
| *Yersinia* | 0.013 | 0.000 | 0.000 | 0.000 | 0.000 | 0.000 | 0.257 |
| *Limosilactobacillus* | 0.012 | 0.000 | 0.000 | 0.003 | 0.000 | 0.000 | 0.313 |
| *Erysipelotrichaceae_UCG_009* | 0.011 | 0.000 | 0.034 | 0.042 | 0.019 | 0.026 | 0.361 |
| *Escherichia_Shigella* | 0.010 | 0.038 | 0.047 | 0.033 | 0.027 | 0.046 | 0.596 |
| *Erysipelotrichaceae_UCG_008* | 0.010 | 0.000 | 0.000 | 0.070 | 0.029 | 0.040 | **0.008** |
| *Ruminococcus* | 0.010 | 0.284 | 0.121 | 0.034 | 0.030 | 0.010 | 0.420 |
| *Other_Eubacteriales* | 0.010 | 0.000 | 0.000 | 0.000 | 0.000 | 0.000 | 0.257 |
| *Oscillibacter* | 0.010 | 0.000 | 0.000 | 0.004 | 0.000 | 0.006 | 0.816 |
| *Saccharofermentans* | 0.010 | 0.033 | 0.018 | 0.016 | 0.009 | 0.015 | 0.734 |
| *Succinivibrio* | 0.009 | 0.008 | 0.000 | 0.017 | 0.001 | 0.000 | 0.781 |
| *Sharpea* | 0.009 | 0.012 | 0.008 | 0.032 | 0.003 | 0.011 | 0.777 |
| *Eubacterium_siraeum_group* | 0.008 | 0.000 | 0.000 | 0.005 | 0.000 | 0.000 | 0.783 |
| *Other_Paenibacillaceae* | 0.008 | 0.000 | 0.000 | 0.000 | 0.000 | 0.000 | 0.257 |
| *Other_Actinobacteria* | 0.006 | 0.000 | 0.017 | 0.049 | 0.000 | 0.000 | 0.237 |
| *Other_Erysipelatoclostridiaceae* | 0.006 | 0.006 | 0.009 | 0.003 | 0.010 | 0.000 | 0.382 |
| *Parabacteroides* | 0.006 | 0.000 | 0.000 | 0.006 | 0.000 | 0.000 | 0.783 |
| *Acinetobacter* | 0.005 | 0.000 | 0.428 | 0.078 | 0.005 | 0.039 | 0.953 |
| *Other_Kiritimatiellae_WCHB1_41* | 0.005 | 0.003 | 0.000 | 0.004 | 0.000 | 0.000 | 0.393 |
| *Other_Planococcaceae* | 0.005 | 0.000 | 0.000 | 0.205 | 0.000 | 0.000 | 0.927 |
| *Brevibacillus* | 0.002 | 0.000 | 0.000 | 0.011 | 0.000 | 0.000 | 0.587 |
| *Pirellulaceae_p_1088_a5_gut_group* | 0.002 | 0.000 | 0.004 | 0.007 | 0.003 | 0.002 | 0.847 |
| *Fibrobacter* | 0.001 | 0.000 | 0.000 | 0.007 | 0.000 | 0.000 | 0.927 |
| *Other* | 0.001 | 0.016 | 0.006 | 0.006 | 0.001 | 0.004 | 0.828 |
| *Enterococcus* | 0.000 | 0.000 | 0.240 | 0.000 | 0.000 | 0.016 | 0.221 |
| *Other_Enterobacteriaceae* | 0.000 | 0.000 | 0.162 | 0.000 | 0.066 | 0.097 | 0.865 |
| *Eubacterium_tenue_group* | 0.000 | 0.000 | 0.065 | 0.000 | 0.000 | 0.000 | 0.098 |
| *Eubacterium_oxidoreducens_group* | 0.000 | 0.000 | 0.054 | 0.000 | 0.000 | 0.000 | 0.098 |
| *Other_Izemoplasmatales* | 0.000 | 0.000 | 0.052 | 0.013 | 0.000 | 0.052 | 0.953 |
| *Corynebacterium* | 0.000 | 0.006 | 0.025 | 0.148 | 0.022 | 0.065 | **0.021** |
| *Rikenellaceae_dgA_11_gut_group* | 0.000 | 0.084 | 0.016 | 0.063 | 0.038 | 0.088 | 0.175 |
| *Other_Saccharimonadales* | 0.000 | 0.000 | 0.011 | 0.000 | 0.000 | 0.000 | 0.257 |
| *Coprobacter* | 0.000 | 0.000 | 0.010 | 0.010 | 0.000 | 0.006 | 0.698 |
| *Paenibacillus* | 0.000 | 0.000 | 0.009 | 0.022 | 0.000 | 0.005 | 0.444 |
| *Erysipelatoclostridiaceae_UCG_004* | 0.000 | 0.126 | 0.014 | 0.014 | 0.020 | 0.012 | 0.543 |

| | | | | | | | |
|---|---|---|---|---|---|---|---|
| *Other_Bacteroidales_F082* | 0.000 | 0.000 | 0.016 | 0.027 | 0.011 | 0.011 | 0.534 |
| *Eggerthellaceae_DNF00809* | 0.000 | 0.000 | 0.022 | 0.019 | 0.010 | 0.021 | 0.382 |
| *Prevotella_7* | 0.000 | 0.000 | 0.006 | 0.000 | 0.000 | 0.000 | 0.257 |
| *Enterobacter* | 0.000 | 0.013 | 0.010 | 0.002 | 0.000 | 0.002 | 0.221 |
| *Solibacillus* | 0.000 | 0.000 | 0.003 | 0.626 | 0.000 | 0.000 | 0.927 |
| *Aerococcus* | 0.000 | 0.000 | 0.004 | 0.000 | 0.000 | 0.000 | 0.098 |
| *Other_Alphaproteobacteria* | 0.000 | 0.000 | 0.003 | 0.002 | 0.000 | 0.001 | 0.889 |
| *Alloprevotella* | 0.000 | 0.017 | 0.011 | 0.013 | 0.011 | 0.041 | 0.119 |
| *EMP_G18* | 0.000 | 0.010 | 0.001 | 0.002 | 0.000 | 0.001 | 0.781 |
| *Acetobacter* | 0.000 | 0.000 | 0.002 | 0.000 | 0.001 | 0.002 | 0.889 |
| *Other_Succinivibrionaceae* | 0.000 | 0.000 | 0.000 | 0.008 | 0.015 | 0.026 | 0.052 |
| *Rikenella* | 0.000 | 0.000 | 0.000 | 0.006 | 0.000 | 0.000 | 0.378 |
| *Lentilactobacillus* | 0.000 | 0.011 | 0.004 | 0.000 | 0.000 | 0.021 | 1.000 |
| *Ruminococcaceae_CAG_352* | 0.000 | 0.000 | 0.000 | 0.000 | 0.016 | 0.018 | 0.198 |
| *Gordonibacter* | 0.000 | 0.000 | 0.000 | 0.017 | 0.010 | 0.000 | 0.198 |
| *Legionella* | 0.000 | 0.000 | 0.000 | 0.002 | 0.000 | 0.002 | 0.198 |
| *Marvinbryantia* | 0.000 | 0.000 | 0.000 | 0.062 | 0.000 | 0.004 | 0.103 |
| *Lachnoclostridium* | 0.000 | 0.951 | 0.000 | 0.055 | 0.000 | 0.501 | 0.531 |
| *Defluviitaleaceae_UCG_011* | 0.000 | 0.000 | 0.000 | 0.000 | 0.001 | 0.002 | 0.198 |
| *Falsochrobactrum* | 0.000 | 0.000 | 0.000 | 0.002 | 0.000 | 0.000 | 0.378 |
| *Pseudomonas* | 0.000 | 0.000 | 0.000 | 0.000 | 0.000 | 0.000 | 1.000 |
| *Listeria* | 0.000 | 0.000 | 0.000 | 0.000 | 0.000 | 0.000 | 1.000 |
| *Other_Gammaproteobacteria* | 0.000 | 0.000 | 0.000 | 0.022 | 0.000 | 0.000 | 0.378 |
| *Saccharopolyspora* | 0.000 | 0.000 | 0.000 | 0.001 | 0.000 | 0.000 | 0.378 |
| *Lachnospiraceae_NK4A136_group* | 0.000 | 0.000 | 0.000 | 0.000 | 0.075 | 0.000 | 0.378 |
| *Succinivibrionaceae_UCG_001* | 0.000 | 0.000 | 0.000 | 0.001 | 0.000 | 0.000 | 0.378 |
| *Sporobacter* | 0.000 | 0.000 | 0.000 | 0.026 | 0.000 | 0.000 | 0.378 |
| *Oscillospiraceae_V9D2013_group* | 0.000 | 0.000 | 0.147 | 0.000 | 0.000 | 0.000 | 0.257 |
| *Lachnospiraceae_FCS020_group* | 0.000 | 0.000 | 0.000 | 0.002 | 0.000 | 0.000 | 0.378 |
| *Other_Rhizobiaceae* | 0.000 | 0.000 | 0.000 | 0.008 | 0.000 | 0.000 | 0.378 |
| *Other_Burkholderiales* | 0.000 | 0.060 | 0.000 | 0.000 | 0.000 | 0.000 | 0.257 |
| *Ruminiclostridium* | 0.000 | 0.049 | 0.000 | 0.004 | 0.047 | 0.005 | 0.162 |
| *Other_Flavobacteriales* | 0.000 | 0.000 | 0.000 | 0.004 | 0.000 | 0.001 | 0.198 |
| *Prevotellaceae_Ga6A1_group* | 0.000 | 0.144 | 0.004 | 0.025 | 0.000 | 0.004 | 0.677 |
| *Other_Veillonellales_Selenomonadales* | 0.000 | 0.000 | 0.000 | 0.065 | 0.000 | 0.000 | 0.198 |
| *Other_Rhodospirillales* | 0.000 | 0.000 | 0.006 | 0.016 | 0.000 | 0.000 | 0.444 |
| *Caproiciproducens* | 0.000 | 0.000 | 0.000 | 0.000 | 0.057 | 0.065 | 0.198 |
| *Other_Pasteurellaceae* | 0.000 | 0.000 | 0.000 | 0.000 | 0.000 | 0.000 | 1.000 |
| *Weissella* | 0.000 | 0.002 | 0.000 | 0.000 | 0.000 | 0.000 | 0.257 |
| *Frisingicoccus* | 0.000 | 0.000 | 0.005 | 0.000 | 0.000 | 0.000 | 0.257 |
| *Phascolarctobacterium* | 0.000 | 0.020 | 0.000 | 0.000 | 0.007 | 0.000 | 0.927 |
| *Pygmaiobacter* | 0.000 | 0.035 | 0.000 | 0.012 | 0.003 | 0.007 | 0.531 |
| *Parvibacter* | 0.000 | 0.000 | 0.012 | 0.058 | 0.025 | 0.000 | 0.221 |
| *Staphylococcus* | 0.000 | 0.000 | 0.000 | 0.000 | 0.000 | 0.000 | 1.000 |

| | | | | | | | |
|---|---|---|---|---|---|---|---|
| *Mycobacterium* | 0.000 | 0.000 | 0.002 | 0.003 | 0.000 | 0.000 | 0.927 |
| *Psychrobacillus* | 0.000 | 0.000 | 0.000 | 0.010 | 0.000 | 0.000 | 0.378 |
| *Erysipelotrichaceae_UCG_006* | 0.000 | 0.000 | 0.000 | 0.012 | 0.000 | 0.000 | 0.378 |
| *Other_Microbacteriaceae* | 0.000 | 0.000 | 0.005 | 0.020 | 0.120 | 0.000 | 0.587 |
| *Negativibacillus* | 0.000 | 0.000 | 0.031 | 0.015 | 0.000 | 0.022 | 0.816 |
| *Other_Oscillospirales_UCG_011* | 0.000 | 0.000 | 0.037 | 0.000 | 0.031 | 0.050 | 0.382 |
| *Planococcus* | 0.000 | 0.000 | 0.000 | 0.076 | 0.000 | 0.000 | 0.378 |
| *Pseudobutyrivibrio* | 0.000 | 0.000 | 0.000 | 0.115 | 0.000 | 0.134 | 0.103 |
| *Coprobacillus* | 0.000 | 0.000 | 0.000 | 0.003 | 0.000 | 0.000 | 0.378 |
| *Other_Paludibacteraceae* | 0.000 | 0.021 | 0.000 | 0.000 | 0.000 | 0.000 | 0.257 |
| *Lactobacillus* | 0.000 | 0.010 | 0.000 | 0.000 | 0.000 | 0.001 | 0.783 |
| *Lachnospira* | 0.000 | 0.000 | 0.000 | 0.000 | 0.021 | 0.000 | 0.378 |
| *Butyricicoccus* | 0.000 | 0.000 | 0.057 | 0.000 | 0.034 | 0.072 | 0.531 |
| *Oribacterium* | 0.000 | 0.000 | 0.000 | 0.209 | 0.000 | 0.000 | 0.378 |
| *Akkermansia* | 0.000 | 0.000 | 0.000 | 0.005 | 0.033 | 0.025 | **0.024** |
| *Other_Bacteroidota* | 0.000 | 0.028 | 0.000 | 0.004 | 0.000 | 0.001 | 0.816 |
| *Other_Bacteroidales_UCG_001* | 0.000 | 0.000 | 0.000 | 0.001 | 0.000 | 0.000 | 0.378 |
| *Prevotellaceae_NK3B31_group* | 0.000 | 0.000 | 0.019 | 0.011 | 0.000 | 0.052 | 0.698 |
| *Planomicrobium* | 0.000 | 0.000 | 0.000 | 0.062 | 0.000 | 0.000 | 0.378 |
| *Aeriscardovia* | 0.000 | 0.000 | 0.000 | 0.009 | 0.000 | 0.000 | 0.378 |
| *Lachnospiraceae_AC2044_group* | 0.000 | 0.028 | 0.000 | 0.006 | 0.000 | 0.000 | 0.783 |
| *Aerosphaera* | 0.000 | 0.000 | 0.000 | 0.001 | 0.000 | 0.000 | 0.378 |
| *Other_Spirochaetaceae* | 0.000 | 0.009 | 0.000 | 0.027 | 0.027 | 0.012 | **0.031** |
| *Tuzzerella* | 0.000 | 0.011 | 0.000 | 0.000 | 0.000 | 0.000 | 0.257 |
| *Psychrobacter* | 0.000 | 0.000 | 0.000 | 0.055 | 0.000 | 0.000 | 0.378 |
| *Candidatus_Soleaferrea* | 0.000 | 0.000 | 0.000 | 0.014 | 0.000 | 0.000 | 0.378 |
| *Burkholderia_Caballeronia_Paraburkholderia* | 0.000 | 0.000 | 0.000 | 0.000 | 0.000 | 0.043 | 0.378 |
| *Other_Butyricicoccaceae* | 0.000 | 0.000 | 0.000 | 0.057 | 0.000 | 0.000 | 0.198 |
| *Candidatus_Profftella* | 0.000 | 0.000 | 0.000 | 0.005 | 0.000 | 0.000 | 0.378 |
| *Anaerovoracaceae_S5_A14a* | 0.000 | 0.000 | 0.000 | 0.000 | 0.000 | 0.020 | 0.378 |
| *Anaerovibrio* | 0.000 | 0.000 | 0.000 | 0.011 | 0.000 | 0.003 | 0.198 |
| *Other_Peptococcaceae* | 0.000 | 0.000 | 0.000 | 0.005 | 0.000 | 0.000 | 0.378 |
| *Other_Proteobacteria* | 0.000 | 0.000 | 0.000 | 0.003 | 0.000 | 0.002 | 0.198 |
| *Arcanobacterium* | 0.000 | 0.000 | 0.000 | 0.057 | 0.000 | 0.000 | 0.378 |
| *Lysinibacillus* | 0.000 | 0.010 | 0.000 | 0.194 | 0.011 | 0.000 | 0.078 |
| *Other_Burkholderiaceae* | 0.000 | 0.000 | 0.030 | 0.000 | 0.000 | 0.048 | 0.927 |
| *Succiniclasticum* | 0.000 | 0.000 | 0.000 | 0.001 | 0.000 | 0.000 | 0.378 |
| *Coriobacteriaceae_UCG_002* | 0.000 | 0.000 | 0.000 | 0.002 | 0.000 | 0.010 | 0.103 |
| *Clostridium_sensu_stricto_6* | 0.000 | 0.000 | 0.009 | 0.000 | 0.000 | 0.020 | 0.927 |
| *Streptomyces* | 0.000 | 0.000 | 0.000 | 0.000 | 0.004 | 0.000 | 0.378 |
| *Other_Ethanoligenenaceae* | 0.000 | 0.000 | 0.000 | 0.015 | 0.000 | 0.017 | 0.198 |
| *Coriobacteriaceae_UCG_003* | 0.000 | 0.000 | 0.000 | 0.133 | 0.073 | 0.000 | 0.103 |
| *Agathobacter* | 0.000 | 0.035 | 0.000 | 0.026 | 0.022 | 0.000 | 0.587 |
| *Slackia* | 0.000 | 0.000 | 0.000 | 0.002 | 0.000 | 0.000 | 0.378 |

| | | | | | | | |
|---|---|---|---|---|---|---|---|
| *Mucinivorans* | 0.000 | 0.120 | 0.000 | 0.016 | 0.000 | 0.000 | 0.783 |
| *Other_Bacillales* | 0.000 | 0.000 | 0.000 | 0.107 | 0.000 | 0.000 | 0.378 |
| *Ruminococcaceae_UCG_001* | 0.000 | 0.118 | 0.000 | 0.000 | 0.000 | 0.000 | 0.257 |
| *Lachnospiraceae_UCG_002* | 0.000 | 0.000 | 0.000 | 0.000 | 0.000 | 0.017 | 0.378 |
| *Other__Lachnospiraceae* | 0.000 | 0.000 | 0.000 | 0.009 | 0.000 | 0.000 | 0.378 |
| *Other_Chitinophagaceae* | 0.000 | 0.000 | 0.393 | 0.000 | 0.000 | 0.000 | 0.257 |
| *Other_Bacteroidales_p_251_o5* | 0.000 | 0.000 | 0.004 | 0.013 | 0.003 | 0.003 | 0.444 |
| *Lachnospiraceae_UCG_007* | 0.000 | 0.000 | 0.000 | 0.017 | 0.000 | 0.000 | 0.378 |
| *Spirochaetaceae_GWE2_31_10* | 0.000 | 0.000 | 0.000 | 0.001 | 0.000 | 0.000 | 0.378 |
| *Paracoccus* | 0.000 | 0.000 | 0.002 | 0.000 | 0.003 | 0.000 | 0.927 |
| *Pediococcus* | 0.000 | 0.000 | 0.000 | 0.000 | 0.000 | 0.003 | 0.378 |
| *Caryophanon* | 0.000 | 0.000 | 0.000 | 0.015 | 0.000 | 0.000 | 0.378 |
| *Borrelia* | 0.000 | 0.000 | 0.000 | 0.001 | 0.000 | 0.000 | 0.378 |
| *Methanosphaera* | 0.000 | 0.000 | 0.000 | 0.001 | 0.000 | 0.000 | 0.378 |
| *Curtobacterium* | 0.000 | 0.000 | 0.000 | 0.013 | 0.000 | 0.000 | 0.378 |
| *Other_Propionibacteriaceae* | 0.000 | 0.000 | 0.000 | 0.004 | 0.000 | 0.000 | 0.378 |
| *Other_Corynebacteriales* | 0.000 | 0.000 | 0.003 | 0.000 | 0.000 | 0.000 | 0.257 |
| *Other_Burkholderiales_A21b* | 0.000 | 0.000 | 0.000 | 0.000 | 0.000 | 0.000 | 1.000 |
| *Sphaerochaeta* | 0.000 | 0.000 | 0.000 | 0.001 | 0.000 | 0.000 | 0.378 |
| *Paraeggerthella* | 0.000 | 0.000 | 0.006 | 0.012 | 0.000 | 0.010 | 0.587 |
| *Leucobacter* | 0.000 | 0.000 | 0.000 | 0.000 | 0.008 | 0.000 | 0.378 |
| *Klebsiella* | 0.000 | 0.000 | 0.000 | 0.000 | 0.000 | 0.000 | 1.000 |
| *Other_Coriobacteriales_Incertae_Sedis* | 0.000 | 0.010 | 0.000 | 0.000 | 0.000 | 0.000 | 0.257 |
| *Lactobacillaceae_HT002* | 0.000 | 0.000 | 0.000 | 0.000 | 0.000 | 0.000 | 1.000 |
| *Other_Micrococcaceae* | 0.000 | 0.000 | 0.000 | 0.019 | 0.000 | 0.000 | 0.378 |
| *Other_Enterobacterales* | 0.000 | 0.000 | 0.000 | 0.055 | 0.000 | 0.180 | 0.198 |
| *Ochrobactrum* | 0.000 | 0.000 | 0.000 | 0.002 | 0.000 | 0.000 | 0.378 |
| *Moraxella* | 0.000 | 0.000 | 0.000 | 0.002 | 0.000 | 0.000 | 0.378 |
| *Nocardiopsis* | 0.000 | 0.000 | 0.005 | 0.000 | 0.000 | 0.000 | 0.257 |
| *Other_Bacillaceae* | 0.000 | 0.000 | 0.000 | 0.011 | 0.000 | 0.000 | 0.378 |
| *Shinella* | 0.000 | 0.000 | 0.000 | 0.001 | 0.000 | 0.000 | 0.378 |
| *Other_Sphingobacteriaceae* | 0.000 | 0.000 | 0.000 | 0.000 | 0.003 | 0.000 | 0.378 |
